# Preclinical evidence and mechanistic insights of ligustilide in ischemic stroke: a systematic review and meta-analysis

**DOI:** 10.3389/fphar.2025.1666207

**Published:** 2025-11-21

**Authors:** Hengtong An, Shuhan Guo, Wei Wang, Tiangang Zheng, Xiaofei Jin, Xiaohong Zhou, Weijuan Gao

**Affiliations:** 1 College of Integrated Traditional Chinese and Western Medicine, Hebei University of Chinese Medicine, Hebei, China; 2 Science and Technology Achievement Transformation Center, Hebei University of Chinese Medicine, Hebei, China; 3 Hebei Key Laboratory of Chinese Medicine Research on Cardio-Cerebrovascular Disease, Hebei University of Chinese Medicine, Hebei, China

**Keywords:** ligustilide, ischemic stroke, animal model, meta-analysis, mechanism research

## Abstract

**Introduction:**

Ligustilide, a phthalide-derived bioactive compound abundantly found in traditional Chinese medicinal herbs such as *Angelica sinensis* (Danggui) and *Ligusticum chuanxiong* (Chuanxiong), has attracted increasing attention for its potential therapeutic benefits in ischemic stroke (IS). However, its clinical applications remain limited, and the comprehensive preclinical evidence regarding its efficacy and mechanisms of action is still unclear.

**Materials and methods:**

A systematic search of PubMed, Web of Science, and Embase was conducted to identify preclinical studies evaluating the effects of Ligustilide in IS animal models. A meta-analysis was performed to quantitatively assess the efficacy of Ligustilide in reducing infarct volume and improving neurological function. Additional analyses explored its potential mechanisms and the sources of heterogeneity.

**Results:**

The pooled results from 13 studies demonstrated that Ligustilide significantly reduced infarct volume (SMD = 3.26, 95% CI [2.31, 4.22], P < 0.05) and improved neurological scores (SMD = 1.64, 95% CI [1.13, 2.15], P < 0.05) in animal models of IS compared to control groups. Mechanistically, Ligustilide exerted protective effects by alleviating oxidative stress [lowering Malondialdehyde (MDA) levels (n = 3) and enhancing Superoxide Dismutase (SOD) (n = 2) and Glutathione (GSH) (n = 2) levels], suppressing inflammatory responses [reducing Tumor Necrosis Factor-alpha (TNF-α) (n = 3)], and a non-significant trend toward reduced apoptosis was also noted based on TUNEL staining (n = 2, P = 0.054), warranting further investigation. Sensitivity analyses confirmed the robustness of the findings. Subgroup analyses indicated that heterogeneity might be associated with differences in modeling methods, administration routes, and the use of multiple intervention doses.

**Conclusion:**

This systematic review and meta-analysis provides comprehensive preclinical evidence supporting the protective effects of Ligustilide in IS animal models through multi-target mechanisms. Future large-scale, high-quality animal studies and clinical trials are needed to further validate its therapeutic potential and facilitate its translational application.

## Graphical Abstract

**Figure:**
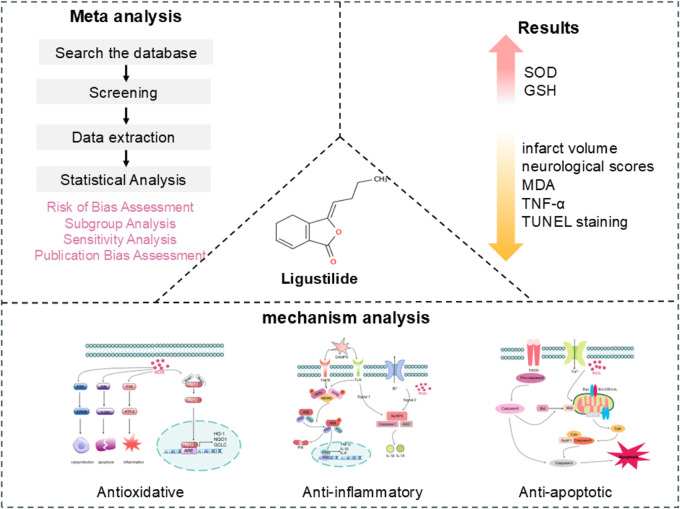

## 1 Background

Stroke represents an acute cerebrovascular disorder marked by an abrupt decline or cessation of cerebral perfusion, resulting in oxygen deprivation and subsequent injury to brain tissue. Pathophysiologically, it is primarily divided into two categories: ischemic stroke (IS), caused by vascular occlusion, and hemorrhagic stroke (HS), resulting from vessel rupture. Common clinical manifestations include mild hemiparesis, dysarthria, sensory deficits, aphasia, and visual disturbances, which pose a significant threat to patients’ lives and markedly impair their quality of life. Worldwide, IS accounts for about 80% of all stroke cases and is the leading cause of death or long-term disability (Virani et al., 2021), and the report of the Global Burden of Disease Study 2019 (GBD 2019) also shows that stroke is a very important disease to be taken seriously as he is the world’s second leading cause of death, and the third leading cause of disability (2021). The most important current therapeutic measures for IS mainly include the use of thrombolytic drugs such as recombinant tissue-type plasminogen activator (rt-PA) and endovascular thrombectomy. These interventions are designed to re-establish cerebral blood flow and enhance neurological recovery (Powers et al., 2019). However, the clinical efficacy of these interventions is limited by a narrow therapeutic time window (typically within 4.5 h of symptom onset), strict eligibility criteria, and the risk of hemorrhagic transformation. Moreover, ischemia-reperfusion injury may occur following revascularization, driven by secondary inflammatory responses and oxidative stress, which further exacerbate neuronal damage and worsen prognosis (Chamorro et al., 2016). Therefore, there is an urgent need to develop neuroprotective strategies targeting multiple pathological mechanisms to complement and enhance current treatment modalities.

**FIGURE 1:**
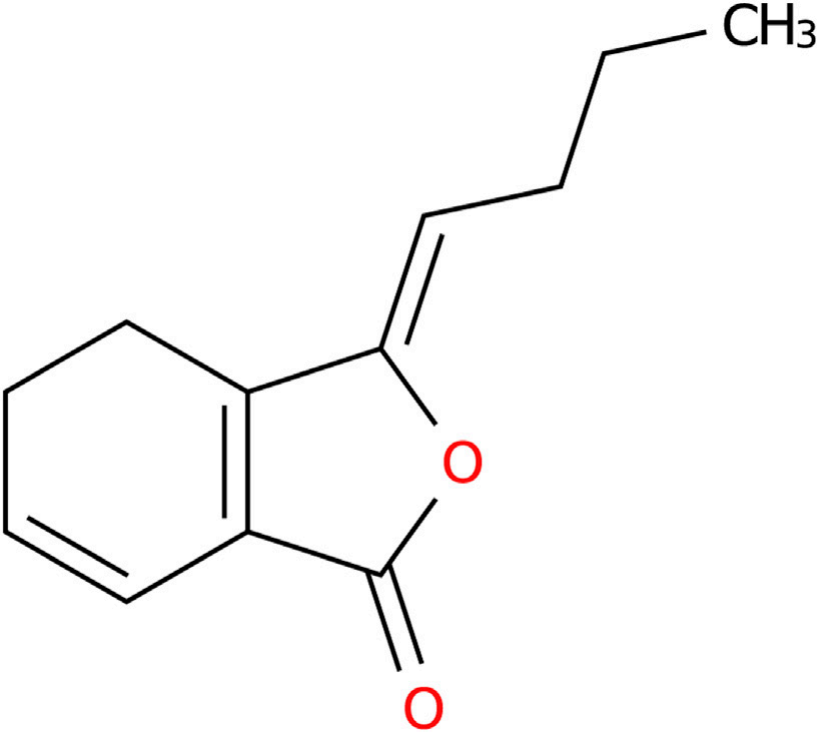
The structural formula of Ligustilide.

Ligustilide is a phthalolactone active ingredient extracted from the herbs Angelica sinensis and Rhizoma Ligusticum chuanxiong, which are commonly used in traditional Chinese medicine. It has the molecular formula C_12_H_14_O_2_ (Figure 1), and is characterized by its high lipophilicity, favorable blood-brain barrier permeability, and multi-target pharmacological activity (Xie et al., 2020). Extensive evidence from various models of neurological diseases has demonstrated that Ligustilide exerts neuroprotective effects through multiple mechanisms, including anti-inflammatory, antioxidant (Peng et al., 2024), anti-apoptotic (Yao et al., 2024), and mitochondrial function-improving properties (Wu et al., 2022a) Notably, in experimental models of IS, Ligustilide has shown prominent efficacy, significantly reducing infarct volume and improving neurological scores.

However, research on the therapeutic effects of Ligustilide in IS remains in the preclinical stage. Due to variations in animal strains, modeling approaches, dosing regimens, timing of intervention, and laboratory settings across studies, the consistency and reliability of existing results remain uncertain and warrant further systematic evaluation and quantitative synthesis.

Previous preclinical meta-analyses have investigated other neuroprotective agents of natural origin, such as astragaloside IV and ligustrazine, which demonstrated significant improvements in neurological function, infarct volume, and inflammatory or oxidative stress markers in ischemic stroke models (Wang et al., 2024; Wang et al., 2017). However, these studies were limited to specific compound classes (saponins and alkaloids) and did not incorporate comprehensive mechanistic biomarkers or meta-regression analyses to explore heterogeneity. Moreover, none of them addressed pharmacokinetic or delivery-related limitations that often hinder the translational potential of volatile phthalide compounds like Ligustilide.

In contrast, the present study provides the first preclinical meta-analysis focusing on Ligustilide, a representative phthalide compound derived from Angelica sinensis and Ligusticum chuanxiong. By integrating both efficacy outcomes (neurological function, infarct volume) and mechanistic indices (oxidative stress, inflammation, apoptosis), and further applying meta-regression to identify potential dose-response and methodological influences, this work extends beyond prior analyses. Furthermore, it discusses pharmacokinetic and formulation strategies that may enhance Ligustilide’s bioavailability and brain delivery, thereby offering new translational insights.

## 2 Methods

### 2.1 Registration

This systematic review and meta-analysis was conducted under the guidance of PRISMA 2020 guidelines (Page et al., 2021), which can ensure the transparency and reproducibility of the research process and the robustness of the research methodology. In addition, the study has been pre-registered on the internationally recognised Register of Systematic Review and Meta-Analysis Protocols (inplasy.com) with the registration code INPLASY202540083.

### 2.2 Search strategy

A systematic literature search was carried out across three prominent biomedical databases—PubMed, Web of Science, and EMBASE—to retrieve preclinical animal studies evaluating the effects of Ligustilide on IS, covering publications from inception through April 2025. To enhance retrieval sensitivity, both controlled vocabulary terms (MeSH/Emtree) and relevant free-text keywords related to “Ligustilide” and “ischemic stroke” were utilized. Additionally, manual screening of reference lists from all included papers was performed to identify any eligible studies that may have been overlooked in the electronic search. The language restriction was set to English. Two independent reviewers conducted the screening process, and any disagreements were resolved through discussion with the corresponding author. Full search algorithms for each database are detailed in the Supplementary Material.

### 2.3 Inclusion and Exclusion Criteria

The eligibility criteria were established based on the PICO framework as follows: Inclusion Criteria (1) Population: Studies employing animal models of IS, including but not limited to models induced by MCAO, filament insertion, or photothrombotic methods to establish focal cerebral ischemia. No restrictions were placed on animal species (e.g., rats, mice), sex, or body weight. (2) Intervention: The experimental group received Ligustilide monotherapy, with no restrictions on the route of administration (e.g., intraperitoneal injection, gavage, tail vein injection) or timing of administration (e.g., pre-treatment, post-ischemic treatment). (3) Comparison: The control group consists of stroke animal models that receive no treatment or are subjected to intervention with a vehicle of equal volume. (4) Outcomes: The primary outcomes included infarct volume and neurological scores. Secondary outcomes comprised oxidative stress markers, inflammatory cytokine expression, and apoptosis-related indicators. Exclusion Criteria (1) Non-animal studies, including clinical trials, *in vitro* experiments, reviews, and commentaries. (2) Studies that met inclusion criteria but lacked full-text availability. (3) Studies involving combination therapy with Ligustilide and other agents, or those lacking an appropriate control group. (4) Duplicate publications. (5) Studies lacking key quantitative data such as means, standard deviations (SD), standard errors (SE), or sample sizes.

### 2.4 Data extraction

Two reviewers independently carried out the data extraction process. In cases of disagreement, consensus was reached through consultation with the corresponding author. The collected information comprised: (1) Year of publication of the article and name of the author (2) Details of the animal model, including species, sex, body weight, age, and the method used to induce IS; (3) Details of the intervention in the administration and control groups, including the dose administered, route of administration, and duration of treatment (4) Primary outcomes: infarct volume and neurological scores; (5) Secondary outcomes: indicators of oxidative stress (MDA, SOD, GSH), inflammatory cytokines (TNF-α), and apoptosis-related markers (TUNEL staining).

As some studies are unable to provide raw data only in graphical form, we will try to ask if the raw data can be made available. If no response was received, data were extracted from figures using WebPlotDigitizer4.5. To ensure accuracy, the same two reviewers independently digitized the graphs and performed cross-checking.

When outcome data were reported as standard error of the mean (SEM), standard deviations (SD) were calculated using the formula: SD = SEM × √n。In studies that reported multiple Ligustilide treatment arms with different dosages, we followed the approach recommended by the Cochrane Handbook for Systematic Reviews of Interventions (CHSRI) (Higgins et al., 2024) to combine these subgroups into a single intervention group for each outcome. The formulas used for pooling continuous variables were as follows:

$$N=\sum_{i=1}^{k}n_{i}$$

$$\bar{X_{\text{combined}}}=\frac{\sum_{i=1}^{k}n_{i}\cdot \bar{X_{i}}}{\sum_{i=1}^{k}n_{i}}$$

$$SD_{\text{combined}}=\sqrt{\frac{\sum_{i=1}^{k}\left(n_{i}-1\right)\cdot SD_{i}^{2}+\sum_{i=1}^{k}n_{i}\cdot {\left(\bar{X_{i}}-\bar{X_{\text{combined}}}\right)}^{2}}{\sum_{i=1}^{k}n_{i}-1}}$$

### 2.5 Risk of Bias assessment

To assess the quality of studies in the included literature, the SYRCLE Risk of Bias tool (Hooijmans et al., 2014) was used for quality assessment. It has been extensively adopted in systematic reviews involving laboratory-based animal models. It comprises ten domains: (1) Sequence generation, (2) Baseline characteristics, (3) Allocation concealment, (4) Random housing, (5) Blinding of caregivers and researchers (performance bias), (6) Random outcome assessment, (7) Blinding of outcome assessors (detection bias), (8) Incomplete outcome data, (9) Selective outcome reporting, (10) Other sources of bias. Each item is scored individually, and an overall risk profile (maximum of 10 points) can be derived for descriptive purposes.

Each item in the study will be rated as low risk, high risk, or unclear based on 10 previously established criteria. This item was assessed independently by two investigators and any disagreements were resolved by negotiation.

### 2.6 Statistical analysis

The statistical analysis of this study was performed using STATA 12.0. software. The effect sizes were expressed as standardized mean difference (SMD) along with 95% confidence intervals. A fixed-effect model was adopted when heterogeneity was low (I^2^ ≤ 50%). In cases of substantial heterogeneity (I^2^ > 50%) or evident methodological differences across studies (e.g., animal species, modeling methods, dosing regimens), a random-effects model was applied to better account for between-study variability. The difference is considered statistically significant when P < 0.05.

### 2.7 Subgroup and meta-regression analysis

To explore potential sources of heterogeneity and the influence of different experimental conditions on the outcomes, predefined subgroup analyses were conducted. The subgroups included: (1) animal species, (2) IS modeling methods, (3) route of administration, and (4) Whether multiple intervention doses exist. Random-effects models were used for all subgroup analyses. The pooled effect size and 95% CI were calculated within each subgroup, and the I^2^ statistic was reported to assess heterogeneity between subgroups. Stratified comparisons were then performed to identify differences in effect estimates.

In addition, meta-regression analyses were conducted using the same four variables to further assess their potential contribution to heterogeneity. A random-effects meta-regression model weighted by the inverse variance was applied.

### 2.8 Publication bias assessment

Publication bias of the included studies was assessed with funnel plots and Egger regression tests. Funnel plots provided a graphical evaluation of symmetry between standard errors and corresponding effect sizes. Additionally, Egger’s linear regression analysis was conducted to statistically detect asymmetry, with a significance level of P < 0.05 suggesting possible bias. If in the presence of publication bias, a trim-and-fill method was used to re-add the missing studies and observe the robustness of the final results.

### 2.9 Sensitivity analysis

A sensitivity analysis of the included studies was conducted using the leave-one-out method, eliminating each piece of literature one by one, and observing whether there would be a significant change in the effect sizes, as a way of determining whether there were any studies that had a disproportionate effect on the overall effect sizes.

### 2.10 Dose–effect relationship analysis

To investigate the potential relationship between Ligustilide dosage and its neuroprotective effects, dose–effect relationship plots were generated for the two primary outcomes—infarct volume and neurological function score—to visually assess the trend in effect sizes across different dose levels.

## 3. Results

### 3.1 Literature search results

**FIGURE 2:**
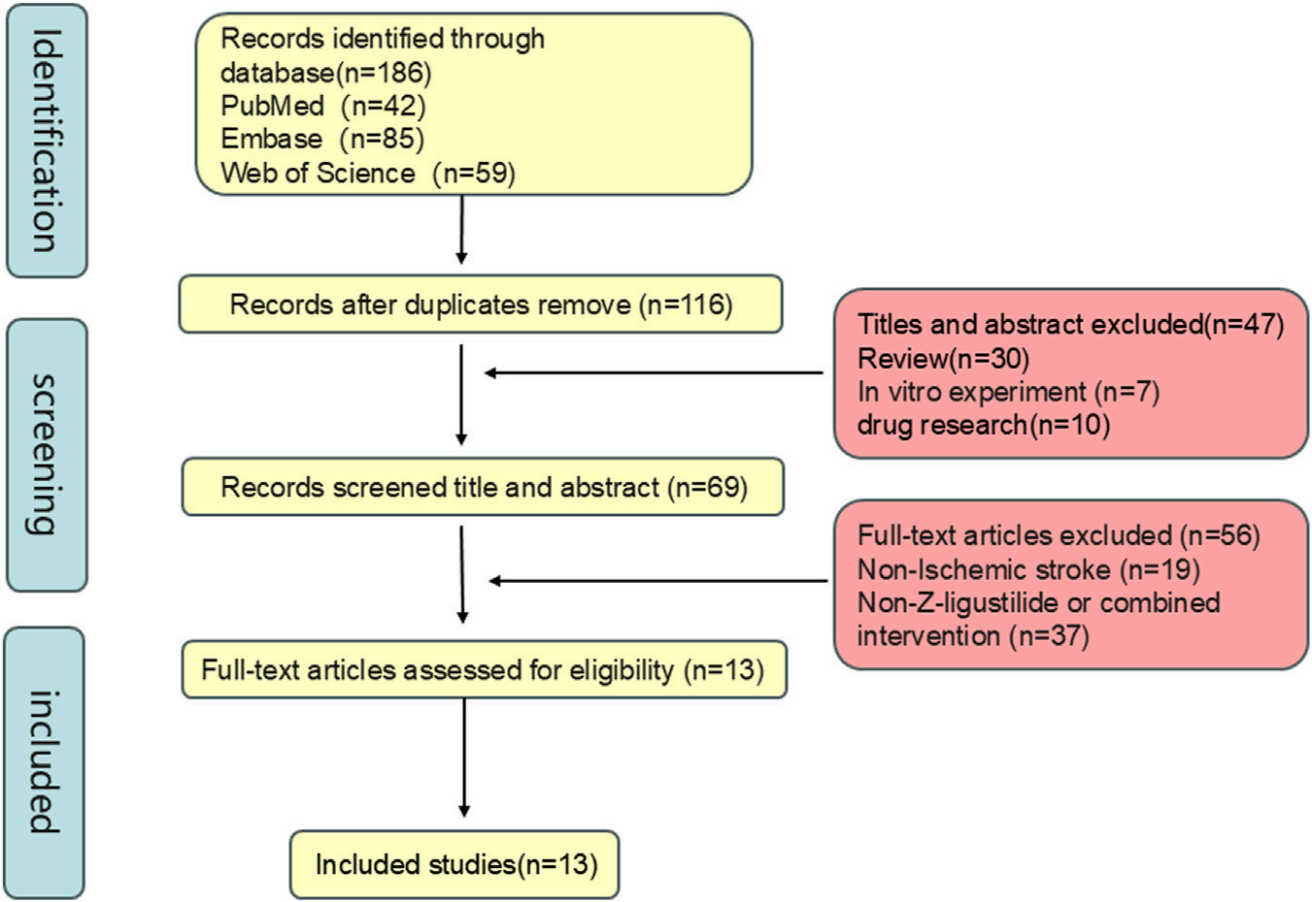
Literature screening Flowchart.

A total of 186 articles were retrieved from the three databases, including 42 from PubMed, 85 from Embase, and 59 from Web of Science. After removing duplicates, 116 articles remained. Title and abstract screening excluded 47 studies based on the following criteria: (1) review articles; (2) *in vitro* experiments; and (3) studies focusing solely on the pharmacological properties of Ligustilide without *in vivo* intervention. Subsequently, full-text screening of the remaining studies led to the exclusion of 56 additional articles for the following reasons: (1) use of non-ischemic stroke animal models; (2) use of Ligustilide in combination with other compounds or interventions rather than as monotherapy. Ultimately, 13 studies met the inclusion criteria and were included in the final systematic review and meta-analysis (Figure 2).

### 3.2 Study characteristics

(1) The publication years of the selected studies ranged from 2006 to 2022. (2) A total of 13 studies (Kuang et al., 2006; Peng et al., 2007; Wu et al., 2011; Peng et al., 2013; Kuang et al., 2014; Yu et al., 2015; Barzegar-Fallah et al., 2016; Li et al., 2017; Long et al., 2018; Ren et al., 2020; Wu et al., 2020; Mao et al., 2022; Wu et al., 2022a),were eligible for inclusion. Among them, nine studies used Sprague-Dawley (SD) rats weighing 230–300g, two studies used C57BL/6 (C57) mice weighing 21–28 g, one study employed Wistar rats (250–280 g), and one study used Institute of Cancer Research (ICR) mice (28–30 g). Due to discrepancies in the number of animals reported for different outcomes, the total sample size was not calculated. (3) All included studies utilized male animals. (4) Regarding model establishment, nine studies used the middle cerebral artery occlusion/reperfusion (MCAO/R) model, two studies used the permanent MCAO model, one study adopted the Forebrain cerebral ischemia (FCI) model, and one study employed the bilateral common carotid artery occlusion (BCCAO) model. (5) With respect to the timing of intervention, nine studies administered Ligustilide postoperatively, three studies applied it as a pretreatment, and one study did not specify the timing. (6) The dosage of Ligustilide ranged from 5 to 80 mg/kg across studies. (7) In terms of administration routes, seven studies employed intraperitoneal injection, three studies used gavage, two studies used nasal administration, and one study applied intravenous injection via the tail vein. (8) For outcome measures, 12 studies reported infarct volume, and nine studies evaluated neurological scores. Additionally, a limited number of studies assessed mechanism-related indicators such as oxidative stress, inflammation, and apoptosis (Table 1).

**TABLE 1:** Basic characteristics of the included studies.

Study ID	Animal characteristics	Animal model	Ligustilide Dosage (mg/kg)	Method of administration	Course	Outcome
Kuang et al. (2006)	Male ICR mouses (28–30 g)	FCI model	5/20	Intraperitoneal injection	Administered at the onset of reperfusion	1.Infarct volume ↓(P < 0.05) 2. Neurological score ↓(P < 0.05) 3. MDA ↓(P < 0.05) 4.GSH ↑(P < 0.05) 5.SOD ↑(P < 0.05)
Peng et al. (2007)	Male SD rats (250–300 g)	MCAO model	20/80	Oral gavage	Administered 2 h post-ischemia	1.Infarct volume ↓(P < 0.05) 2. Neurological score ↓(P < 0.05)
Wu et al. (2011)	Male SD rats (250–270 g)	MCAO/reperfusion model	20/40/80	Oral gavage	Administered at 3 h and 0.5 h prior to surgery	1.Infarct volume ↓(P < 0.05) 2. Neurological score ↓(P < 0.05)
Peng et al. (2013)	Male SD rats (230–260 g)	MCAO/reperfusion model	8/16/32	Tail vein injection	Administered at the onset of reperfusion	1.Infarct volume ↓(P < 0.05) 2. Neurological score ↓ (P < 0.05)
Kuang et al. (2014)	Male SD rats (250–300 g)	MCAO/reperfusion Model	20/40	Intraperitoneal injection	Administered at the onset of reperfusion	1.Infarct volume ↓(P < 0.05) 2. Neurological score ↓(P < 0.05) 3.TNF-α ↓(P < 0.05)
Yu et al. (2015)	Male SD rats (230–260 g)	MCAO/reperfusion Model	7.5/15/30	Nasal feeding	NA	1.Infarct volume ↓(P < 0.05)
Fallah et al. (2016)	Male SD rats (250–280 g)	MCAO model	20	Intraperitoneal injection	Administered 1 h prior to surgery	1.Infarct volume ↓(P < 0.05) 2. Neurological score ↓(P < 0.05) 3.MDA ↓(P < 0.05) 4.GSH ↑(P < 0.05) 5.SOD ↑(P < 0.05) 6.TNF-α ↓(P < 0.05)
Li et al. (2017)	Male SD rats (230–270 g)	MCAO/reperfusion Model	15	Nasal feeding	Administered 3 days prior to surgery	1.Infarct volume ↓(P < 0.05) 2.TUNEL staining ↓(P < 0.05)
Long et al. (2018)	Male C57 mouses (24–28 g)	BCCAO/reperfusion Model	30	Intraperitoneal injection	Administered at 0 h, 24 h, and 48 h post-reperfusion	1. Neurological score ↓(P < 0.05) 2.MDA ↓(P < 0.05) 3. TNF-α ↓(P < 0.05)
Ren et al. (2020)	Male C57 mouses (21–23 g)	MCAO/reperfusion Model	5/10/20	Oral gavage	Administered at the onset of reperfusion	1.Infarct volume ↓(P < 0.05)
Wu et al. (2020)	Male SD rats (240–260 g)	MCAO/reperfusion Model	20	Intraperitoneal injection	Administered at the onset of reperfusion	1.Infarct volume ↓(P < 0.05) 2. Neurological score ↓(P < 0.05)
Wu et al. (2022a)	Male SD rats (240–260 g)	MCAO/reperfusion Model	20	Intraperitoneal injection	Administered at the onset of reperfusion	1.Infarct volume ↓(P < 0.05) 2. Neurological score ↓ (P > 0.05) 3.TUNEL staining ↓(P < 0.05)
Mao et al. (2022)	Male SD rats (240–280 g)	MCAO/reperfusion Model	10/20	Intraperitoneal injection	Administered at the onset of reperfusion	1.Infarct volume ↓(P < 0.05) 2. Neurological score ↓(P < 0.05)

Legend: MDA: malondialdehyde; GSH: glutathione; SOD: superoxide dismutase; TNF-α: Tumor Necrosis Factor-alpha; ↑: The indicator increased after the intervention.; ↓: The indicator decreased after the intervention.

### 3.3 Study quality

**FIGURE 3:**
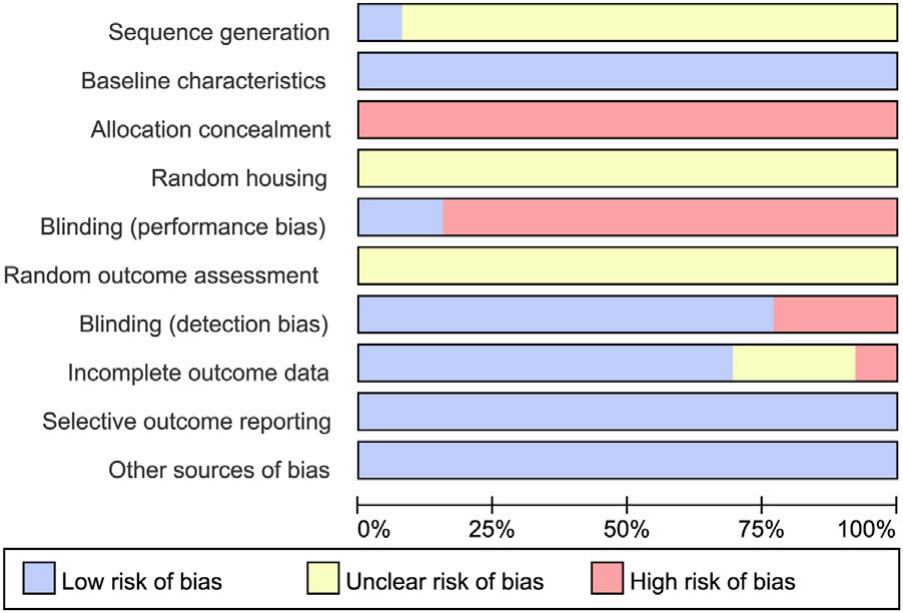
Risk of bias.

The methodological rigor of the 13 included preclinical studies was evaluated utilizing the SYRCLE’s Risk of Bias assessment tool, specifically designed for animal research. As illustrated in Figure 3, none of the studies were rated as low risk across all domains. All studies claimed to have implemented random allocation; however, only one study explicitly described the method of randomization. Baseline characteristics of the animals were reported in all 13 studies. Only two studies reported allocation concealment, and none provided information regarding blinding of personnel during intervention administration (performance bias). Two studies clearly documented the total number of animals used throughout the experiment, whereas the remaining studies did not explicitly report sample size details. None of the studies were prospectively registered. No other potential sources of bias were identified in any of the included studies.

### 3.4 Effectiveness

#### 3.4.1 Primary outcomes

**FIGURE 4:**
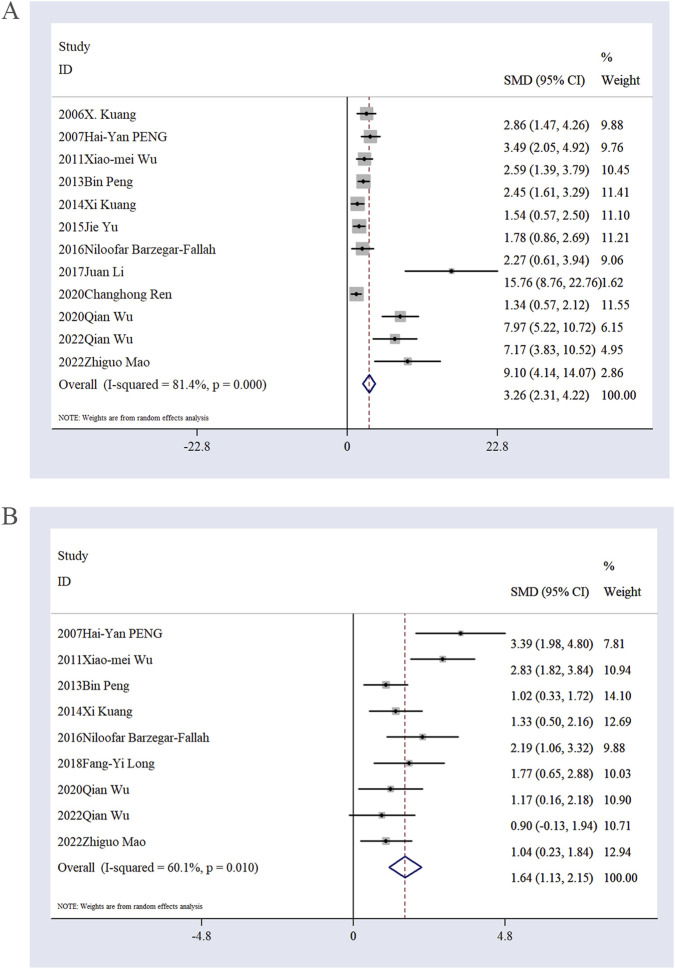
The forest plot of infarct volume **(A)**. The forest plot of neurological score **(B)**.

Effect of Ligustilide on infarct volume percentage. A total of 12 studies (Kuang et al., 2006; Peng et al., 2007; Wu et al., 2011; Peng et al., 2013; Kuang et al., 2014; Yu et al., 2015; Barzegar-Fallah et al., 2016; Li et al., 2017; Long et al., 2018; Ren et al., 2020; Wu et al., 2020; Mao et al., 2022; Wu et al., 2022a) reported data on infarct volume percentage. The pooled results indicated that Ligustilide significantly reduced infarct volume percentage in animal models of IS compared to the model group [n = 267, SMD: 3.26; 95% CI: 2.31, 4.22; P < 0.05; heterogeneity: I^2^ = 81.4%, P < 0.01; Figure 4A].

Effect of Ligustilide on neurological scores. A total of nine studies (Peng et al., 2007; Wu et al., 2011; Peng et al., 2013; Kuang et al., 2014; Barzegar-Fallah et al., 2016; Long et al., 2018; Wu et al., 2020; Mao et al., 2022; Wu et al., 2022a) reported neurological scores. The pooled analysis demonstrated that Ligustilide significantly improved neurological outcomes in animal models of IS compared to the model group [n = 244, SMD: 1.64; 95% CI: 1.13, 2.15; P < 0.05; heterogeneity: I^2^ = 60.1%, P = 0.01; Figure 4B].

#### 3.4.2 Secondary outcomes

**FIGURE 5:**
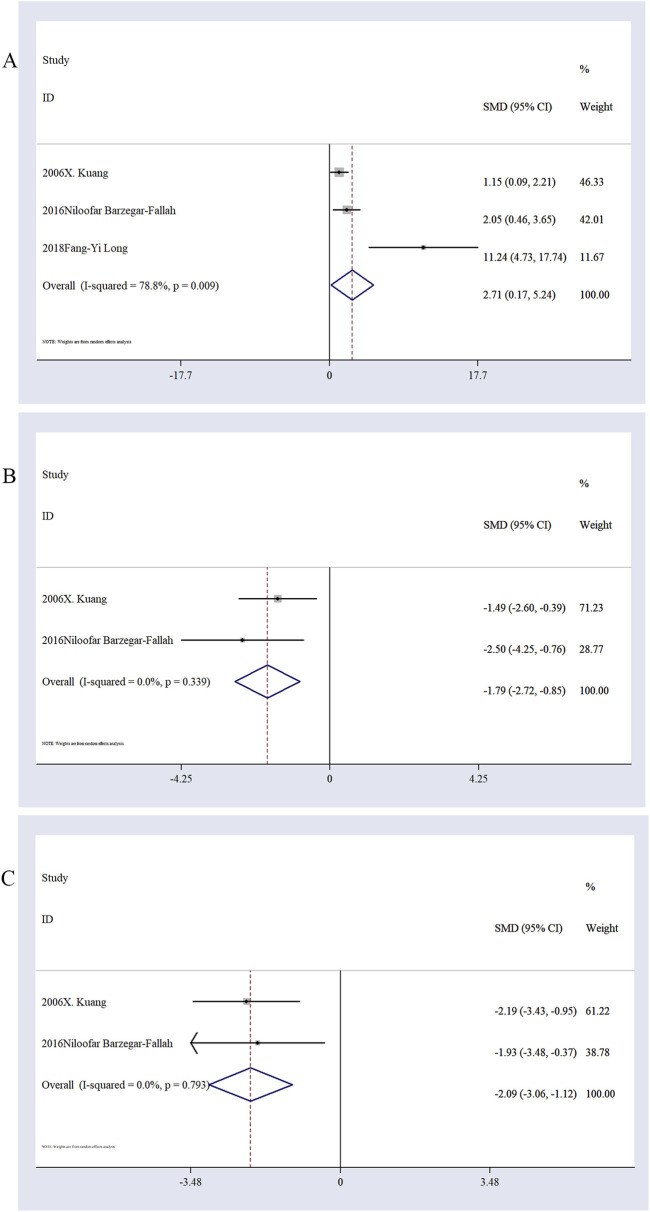
The forest plot of MDA level **(A)**. The forest plot of SOD level **(B)**. The forest plot of GSH level **(C)**.

Effect of Ligustilide on oxidative stress markers. Three studies (Kuang et al., 2006; Barzegar-Fallah et al., 2016; Long et al., 2018) reported changes in malondialdehyde (MDA) levels. Meta-analysis indicated that Ligustilide significantly reduced MDA levels in brain tissue of animal models of IS (n = 36, SMD = 2.71; 95% CI: 0.17, 5.24; P < 0.05; heterogeneity: I^2^ = 78.8%, P < 0.05; Figure 5A). Two studies (Kuang et al., 2006; Barzegar-Fallah et al., 2016) evaluated superoxide dismutase (SOD) levels and suggested a potential increase in SOD content following Ligustilide treatment (n = 28, SMD = −1.79; 95% CI: −2.72, −0.85; P < 0.05; Figure 5B). Although the number of studies is limited, this finding provides preliminary evidence. Similarly, two studies (Kuang et al., 2006; Barzegar-Fallah et al., 2016) reported changes in glutathione (GSH) levels. The pooled results showed a significant increase in GSH levels after Ligustilide intervention (n = 28, SMD = −2.09; 95% CI: −3.06, −1.12; P < 0.05; Figure 5C). However, due to the limited number of studies, this conclusion warrants further validation.

**FIGURE 6:**
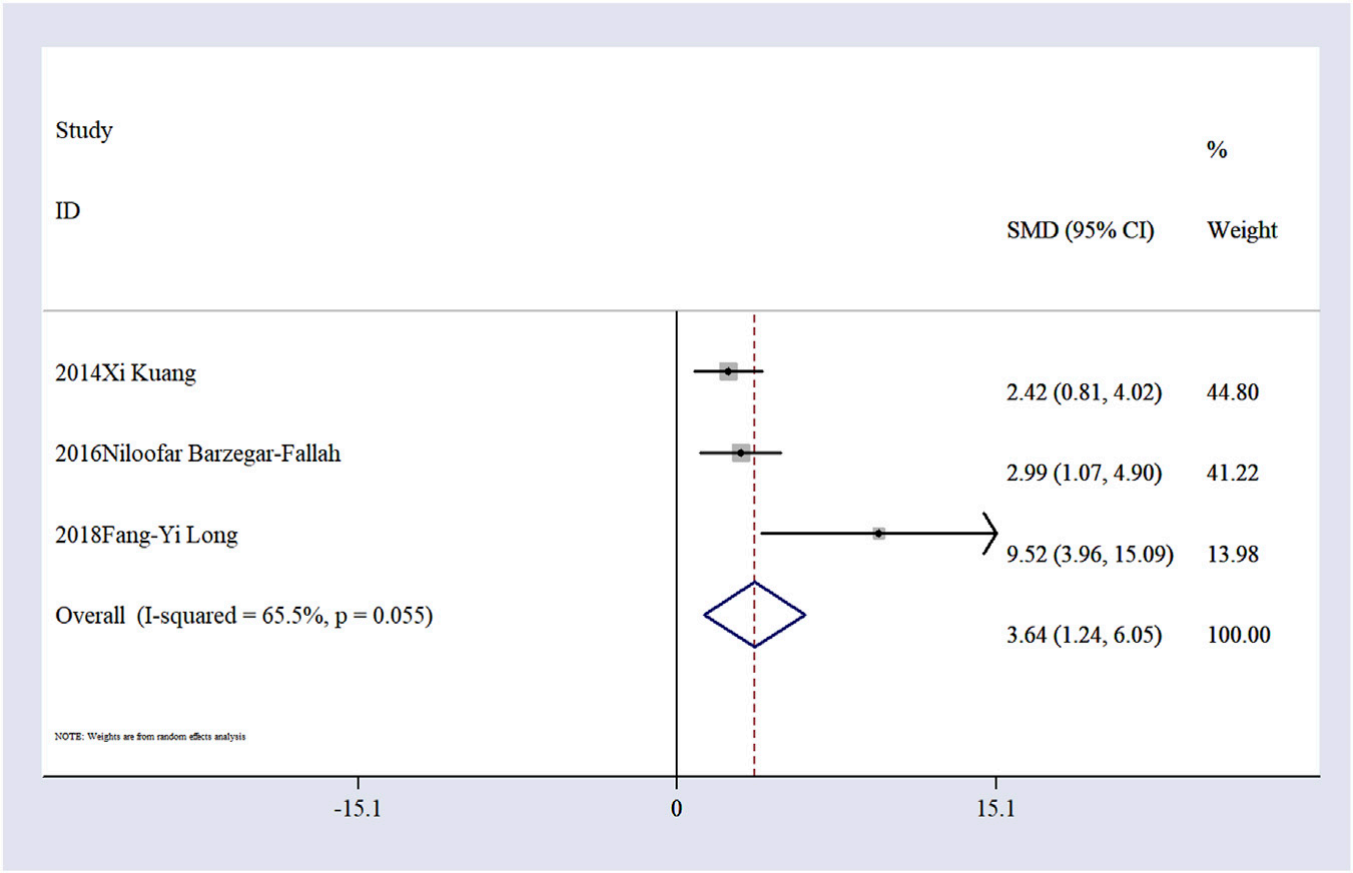
The forest plot of TNF-α level.

Effect of Ligustilide on inflammatory markers. Three studies (Kuang et al., 2014; Barzegar-Fallah et al., 2016; Long et al., 2018) reported the expression levels of tumor necrosis factor-alpha (TNF-α). The results demonstrated that Ligustilide significantly reduced TNF-α expression in animal models of IS (n = 30, SMD = 3.64; 95% CI: 1.24, 6.05; P < 0.05; heterogeneity: I^2^ = 65.5%, P < 0.05; Figure 6).

**FIGURE 7:**
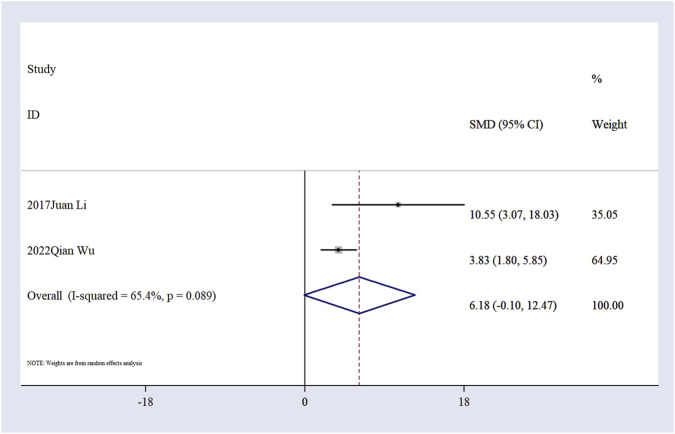
The forest plot of TUNEL staining.

Effect of Ligustilide on apoptosis-related indicators. Two studies (Li et al., 2017; Wu et al., 2022a) evaluated neuronal apoptosis using TUNEL staining. The pooled results indicated a trend toward reduced apoptosis in the Ligustilide-treated group compared to the model group (n = 18, SMD = 6.18; 95% CI: −0.10, 12.47; P = 0.054; Figure 7). Although the effect size suggests a possible anti-apoptotic effect, this result did not reach the conventional threshold for statistical significance. Given the small number of included studies and the wide confidence interval, this evidence remains preliminary and should be interpreted with caution. Further validation through well-designed experimental studies is warranted.

### 3.5 Sensitivity analysis

A leave-one-out sensitivity analysis was conducted for the two primary outcomes (infarct volume and neurological scores). The results indicated that sequential exclusion of any single study did not substantially alter the pooled effect sizes or their 95% confidence intervals. These findings suggest that the conclusions of this meta-analysis are robust and not unduly influenced by any individual study (see Supplementary Figure 1, 2).

### 3.6 Subgroup and meta-regression analysis

In light of the considerable heterogeneity identified in the main outcomes (infarct volume: I^2^ = 81.4%; neurological score: I^2^ = 60.1%), subgroup analyses were conducted to investigate possible sources of variability. The analysis was based on four predefined factors: type of animal used, ischemic model applied, administration route, and whether multiple dosages of the intervention were implemented. The results suggest that the modeling method may be a major contributor to heterogeneity in infarct volume, while both the modeling method, administration route and the presence or absence of multiple intervention doses may account for the heterogeneity in neurological scores (see Supplementary Figure 2, 3; Supplementary Table 1).

To further investigate the potential sources of heterogeneity identified in the subgroup analyses, univariable meta-regression was performed for each of the four predefined factors across the two primary outcomes. However, none of the tested covariates showed statistically significant associations with effect size (all P > 0.05; see Supplementary Tables 2, 3). While subgroup analyses indicated potential effect modification by variables such as modeling method, administration route, and the presence or absence of multiple intervention doses, these associations were not confirmed in the meta-regression. This discrepancy may be attributed to methodological differences between the two approaches, as well as limitations in statistical power. Specifically, several subgroup categories contained only one or two studies, which likely limited the ability of the regression models to detect significant relationships.

### 3.7 Publication bias

**FIGURE 8:**
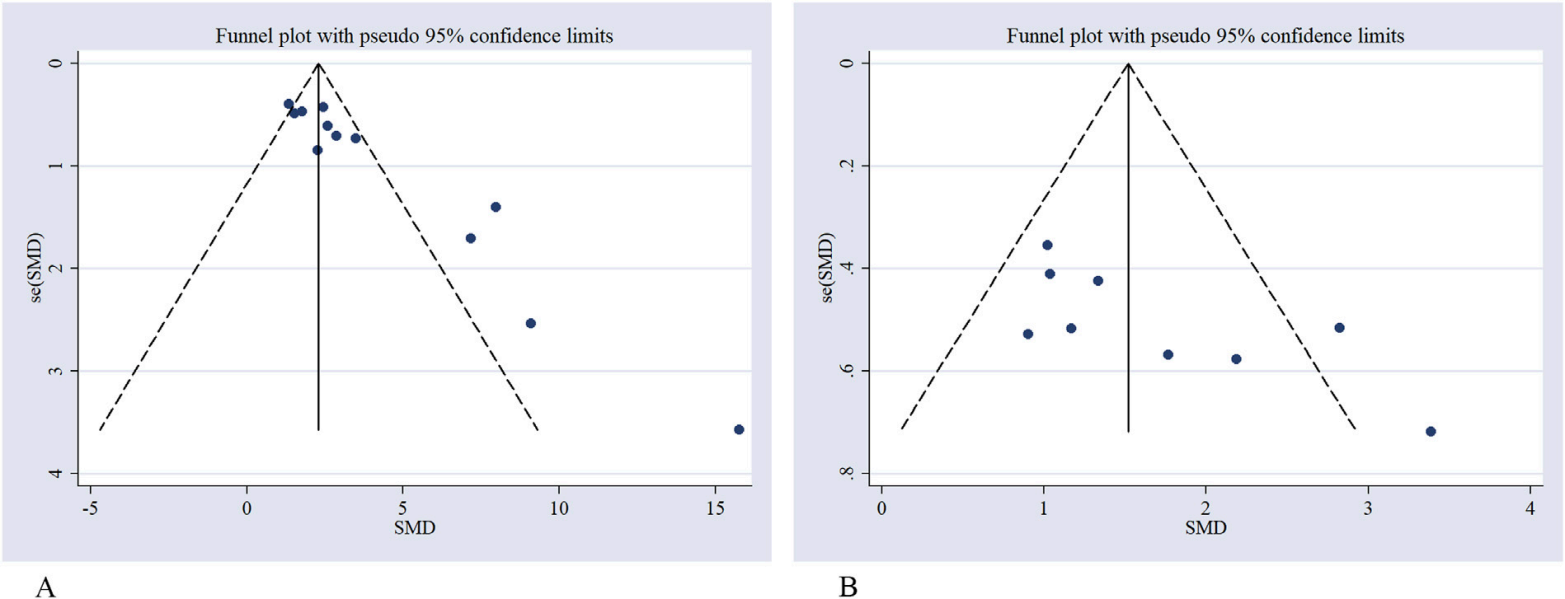
Funnel plot for **(A)** infarct volume, **(B)** neurological score.

**FIGURE 9:**
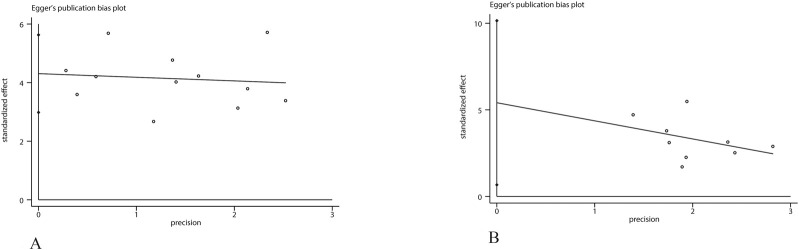
Egger’s publication bias plot for **(A)** infarct volume, **(B)** neurological score.

**FIGURE 10:**
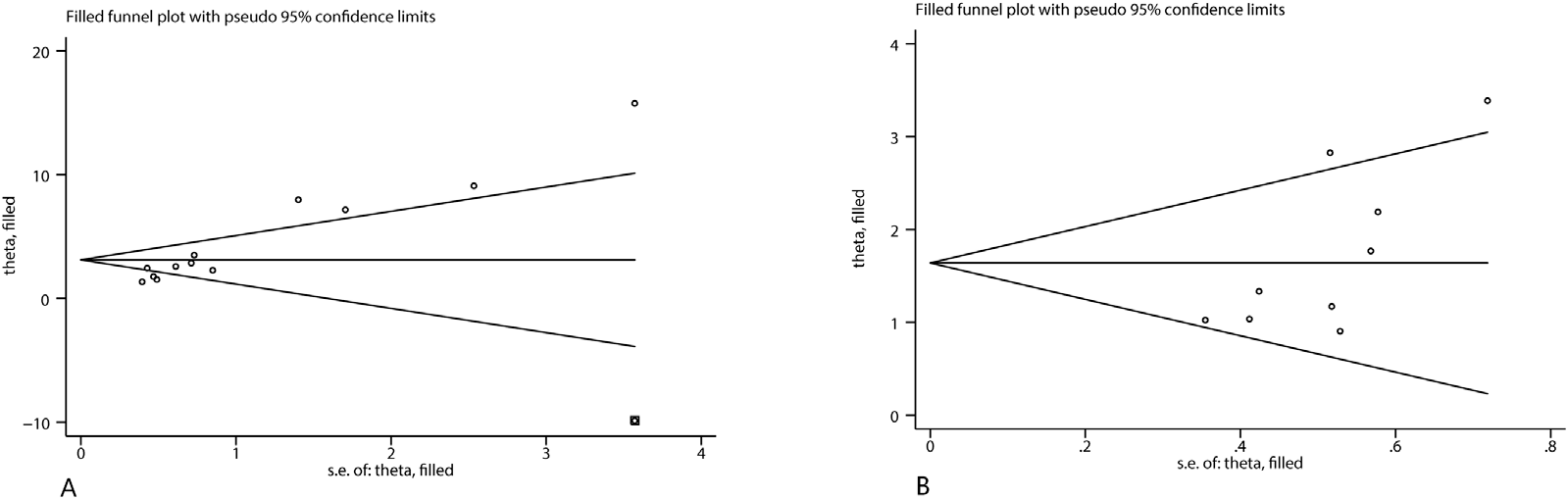
Trim-and-fill analysis for **(A)** infarct volume, **(B)** neurological score.

To assess potential publication bias in the main outcomes, funnel plots were generated. Visual examination indicated an asymmetric distribution, suggesting the possibility of publication bias. This observation was statistically supported by Egger’s regression test (P < 0.05). To further evaluate this, Duval and Tweedie’s trim-and-fill method was applied under a random-effects model. For neurological function outcomes, no trimming or imputation was performed (“no trimming performed; data unchanged”), and the pooled SMD remained 1.64 (95% CI 1.13–2.15). For infarct volume, one study was trimmed and one potentially missing study was imputed on the left side of the funnel plot, yielding an adjusted pooled SMD of 3.12 (95% CI 2.10–4.14), compared with the original estimate of 3.26 (95% CI 2.31–4.22). Although the magnitude slightly decreased, the overall effect remained statistically significant, indicating that potential publication bias had minimal impact on the robustness of the findings (Figures 8–10).

### 3.8 Dose–effect relationship analysis

**FIGURE 11:**
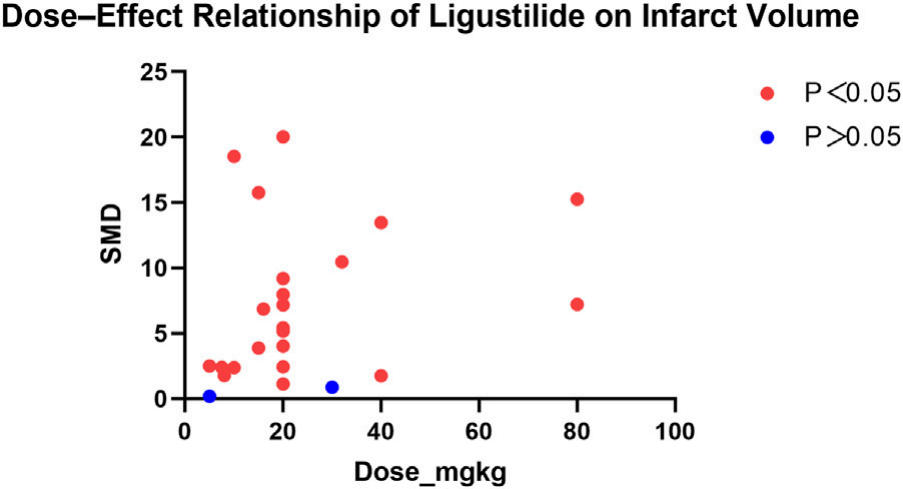
Dose–Effect Relationship of Ligustilide on Infarct Volume (Legend: X-axis: Ligustilide dose (mg/kg); Y-axis: Standardized mean difference (SMD) in infarct volume.).

**FIGURE 12:**
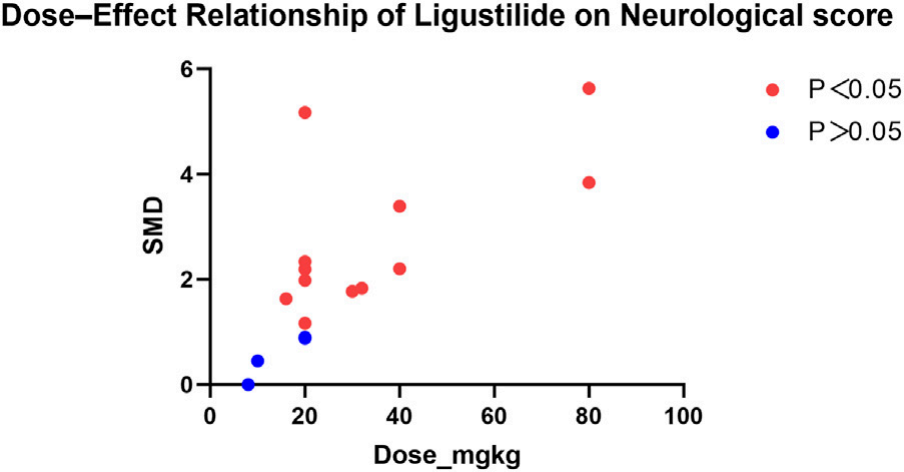
Dose–Effect Relationship of Ligustilide on Neurological score (Legend: X-axis: Ligustilide dose (mg/kg); Y-axis: Standardized mean difference (SMD) in neurological score.).

In this meta-analysis, data from studies reporting multiple Ligustilide dose groups were combined to obtain a single effect estimate per study for pooled analysis. However, to further explore potential associations between Ligustilide dosage and therapeutic efficacy, separate dose–effect scatter plots were generated for the two primary outcomes—infarct volume and neurological function score (Figures 11, 12).

In all included studies, Ligustilide was administered at doses ranging from 5 to 80 mg/kg. The dose–effect scatter plots revealed distinct patterns across the two primary outcomes.

For infarct volume, a clear neuroprotective trend was observed, characterized by a consistent reduction in infarct size relative to the control group. Moderate to high doses (15–40 mg/kg) generally produced larger standardized mean differences (SMDs), indicating stronger neuroprotective efficacy. However, further dose escalation beyond 40 mg/kg did not result in proportionally greater benefits, suggesting the presence of a plateau phase in the dose–response curve.

For neurological function scores, Ligustilide administration within the 5–80 mg/kg range also improved behavioral outcomes compared with controls. Although most doses demonstrated beneficial effects, the relationship between dose and effect size appeared less linear than that observed for infarct volume. Moderate doses (15–30 mg/kg) tended to achieve the greatest improvement in neurological performance, whereas higher doses did not further enhance recovery.

### 3.9 Negative and non-significant findings

Several studies (Wu et al., 2022a) reported non-significant improvements in neurological scores, possibly due to variations in infarct models, administration timing, or measurement scales. These findings highlight the need for standardized preclinical protocols.

## 4 Discussion

### 4.1 Summary of evidence

This systematic review and meta-analysis included a total of 13 preclinical studies and demonstrated that Ligustilide exerts neuroprotective effects in animal models of IS. Analysis of the outcome measures revealed that Ligustilide significantly reduced infarct volume and improved neurological function scores. Although only a limited number of studies evaluated mechanistic outcomes, preliminary evidence suggests that Ligustilide may exert antioxidant effects (e.g., reducing malondialdehyde [MDA] levels and increasing superoxide dismutase [SOD] and glutathione [GSH] levels), and attenuate inflammation (e.g., lowering tumor necrosis factor-alpha [TNF-α] expression). A possible anti-apoptotic effect was also observed based on TUNEL staining results; however, the pooled analysis did not reach the threshold for statistical significance (P = 0.054), and thus this finding should be interpreted with caution and requires further validation in future studies. Notably, substantial heterogeneity was observed in the primary outcomes. Subgroup analyses suggested that modeling methods, administration routes and the presence or absence of multiple intervention doses may contribute to this heterogeneity. Moreover, asymmetry observed in the funnel plot, along with a significant result from Egger’s test, suggested the presence of publication bias. To evaluate its potential influence, the trim-and-fill approach was employed. After imputing the missing studies, the overall effect estimate showed minimal change, indicating that the results of this meta-analysis remain robust despite the likelihood of publication bias.

All included studies reported adherence to local ethical guidelines for animal experimentation. Nonetheless, refinement of experimental design and reduction in animal use remain essential for improving reproducibility and ethical compliance in preclinical stroke research.

### 4.2 Mechanistic insights

A systematic review of the included studies suggests that Ligustilide may exert neuroprotective effects in IS through multiple mechanisms, including antioxidative activity, anti-inflammatory responses, and the inhibition of neuronal apoptosis.

### 4.3 Antioxidative mechanism

Oxidative stress is a pathological state in which the body generates excessive reactive oxygen species and reactive nitrogen beyond the body’s ability to remove them when subjected to a variety of environmental stimuli *in vivo* or *in vitro*, resulting in a dysregulation of intracellular redox balance. Key ROS, including superoxide anion (O_2_^−^), hydrogen peroxide (H_2_O_2_), and hydroxyl radicals (·OH), exhibit high chemical reactivity and can inflict damage on lipids, proteins, and nucleic acids (Orellana-Urzúa et al., 2020). The cellular antioxidant defense comprises both enzymatic components—such as SOD, catalase (CAT), and glutathione peroxidase (GPx)—and non-enzymatic molecules like GSH, which function synergistically to preserve redox equilibrium within the intracellular environment (Pisoschi and Pop, 2015)**.**

In IS, oxidative stress is a well-recognized contributor to secondary brain injury. The interruption of cerebral blood flow leads to oxygen and glucose deprivation, impaired mitochondrial function, calcium overload, and excitotoxicity (Liu et al., 2018). Upon reperfusion, a rapid influx of oxygen into the ischemic area triggers a burst of ROS generation, causing additional damage known as “reperfusion injury” (Andrabi et al., 2020; Turrens, 2003). ROS can compromise blood-brain barrier integrity, induce cerebral edema (Abramov et al., 2007), and initiate lipid peroxidation, forming cytotoxic byproducts such as MDA and 4-hydroxynonenal (4-HNE), which exacerbate neuronal injury (Abarikwu et al., 2012). Studies suggest that Ligustilide may mitigate these effects by reducing ROS accumulation and alleviating oxidative stress–related damage (Xiao et al., 2015; Wu et al., 2022a; Yao et al., 2024).

**FIGURE 13:**
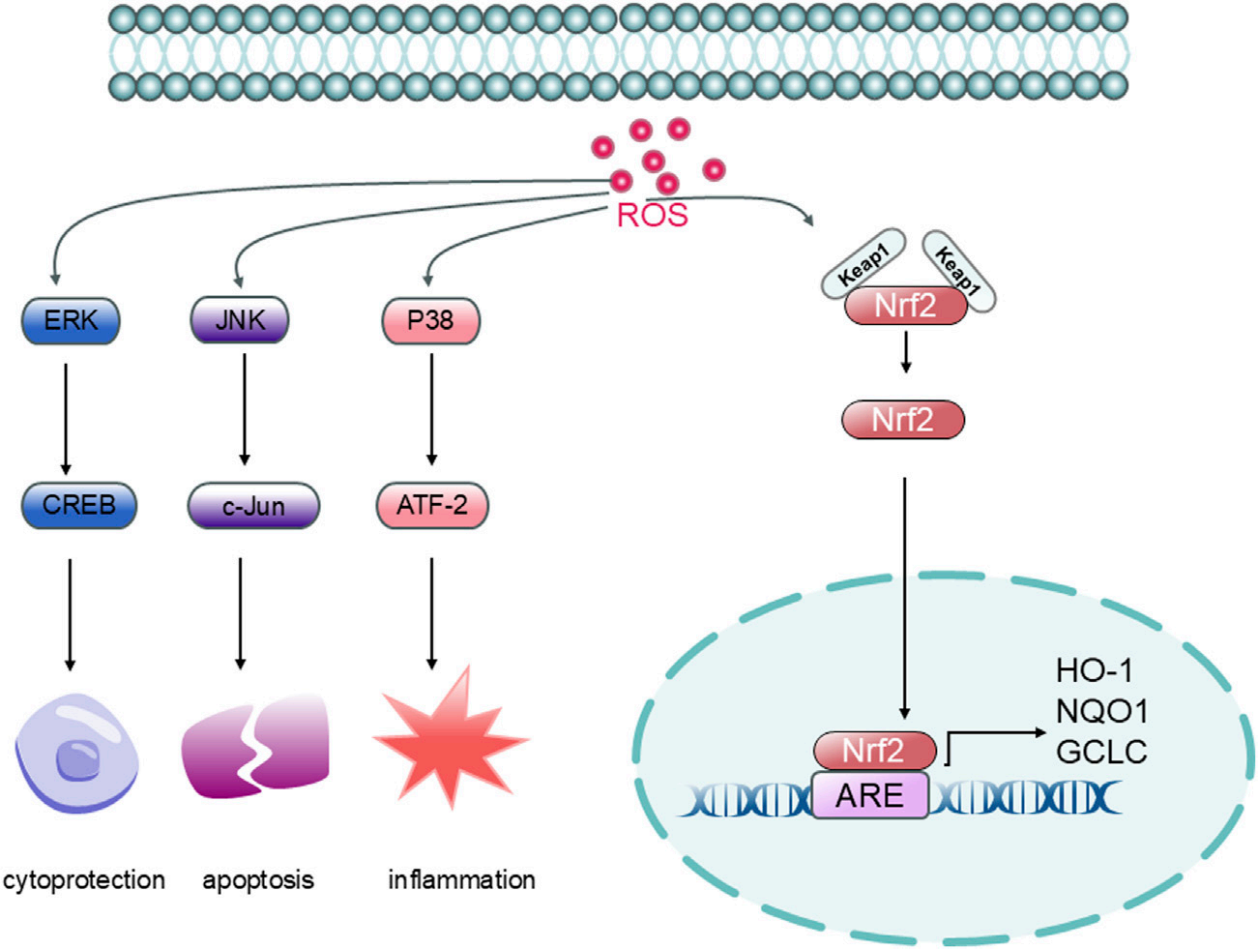
Oxidative Stress Mechanism Diagram (Legend: ROS: Reactive Oxygen Species; Keap1: Kelch-like ECH-associated protein 1; Nrf2: Nuclear factor erythroid 2-related factor 2; ARE: Antioxidant Response Element; HO-1: Heme Oxygenase-1; NQO1: NAD(P)H Quinone Dehydrogenase 1; GCLC: Glutamate-Cysteine Ligase Catalytic Subunit; ERK: Extracellular signal-regulated kinase; CREB: cAMP Response Element-Binding protein; JNK: c-Jun N-terminal Kinase; c-Jun: Jun proto-oncogene; P38: p38 Mitogen-Activated Protein Kinase; ATF-2: Activating Transcription Factor 2)

The effects of oxidative stress on stroke are regulated by several signaling pathways (Figure 13), of which the Nuclear factor erythroid 2-related factor 2/Antioxidant response element (Nrf2/ARE) pathway is the most classically regulated. Under physiological conditions, the inhibitory protein Kelch-like ECH-associated protein 1 (Keap1) specifically binds to Nrf2 and contributes to its degradation. When oxidative stress occurs, the structure of Keap1 is altered, allowing Nrf2 to dissociate from Keap1 and undergo successful nuclear displacement. Nrf2 can bind to ARE sequences in the nucleus, promoting the expression of genes related to anti-oxidative stress, such as Heme oxygenase-1 (HO-1), NAD(P)H quinone oxidoreductase 1 (NQO1), and Glutamate-cysteine ligase catalytic subunit (GCLC) (Kopacz et al., 2020). In addition, the Phosphoinositide 3-kinase/Protein kinase B (PI3K/Akt) pathway is also an important signaling pathway that regulates oxidative stress, and Akt can promote Nrf2 stabilization and enhance the antioxidant response by inhibiting the expression of Glycogen synthase kinase-3 beta (GSK-3β). Both *in vivo* and *in vitro* studies have found that natural products such as ligustilide can promote Nrf2 nuclear translocation, enhance the activity of this pathway, and alleviate oxidative stress (Choi et al., 2018; Peng et al., 2024).

The Mitogen-activated protein kinase (MAPK) signaling pathway is also an important pathway in the regulation of oxidative stress, which includes Extracellular signal-regulated kinase (ERK), c-Jun N-terminal kinase (JNK) and p38 mitogen-activated protein kinase (p38) isoforms. While ERK is generally associated with cell survival and repair, JNK and p38 are involved in pro-apoptotic and inflammatory processes. ROS serves as a common upstream activator of MAPK signaling in ischemic injury. Ligustilide has been shown to modulate MAPK pathways, thereby attenuating neuronal damage and promoting tissue repair (Sun et al., 2021; Chang et al., 2022).

Experimentally, oxidative damage is commonly assessed using markers such as MDA (a product of lipid peroxidation) and antioxidant enzymes like SOD, CAT, and GSH. Several studies have reported that ligustilide significantly improves these oxidative stress-related parameters, thereby exerting antioxidant effects (Peng et al., 2022b; Shi et al., 2023; Sun et al., 2024).

### 4.4 Anti-inflammatory mechanisms

Inflammation is a complex biological response triggered by infection, trauma, or ischemia, aimed at eliminating harmful stimuli and initiating tissue repair. It typically involves immune cell recruitment, cytokine release, and increased vascular permeability. While acute inflammation is protective, dysregulated or sustained inflammatory responses can cause secondary damage to host tissues.

After an IS occurs, it triggers a series of secondary injuries in which inflammation plays an important role anyway. When brain ischemia occurs, cellular metabolism is disturbed, ionic imbalance, and mitochondrial damage, resulting in the release of a large number of danger-associated molecular patterns (DAMPs) (Fu et al., 2015; Gadani et al., 2015), which can be activated by pattern-recognition receptors (PRRs) on the surface of immune cells, including Toll-like receptors (TLRs) and NOD-like receptors (NLRs), thus activating inflammatory cascade reactions and causing various types of Immune cells are recruited to the lesion, forming a local immune microenvironment (Kleinschnitz et al., 2013; Mracsko et al., 2014; Shi et al., 2018). Inflammatory cells release pro-inflammatory cytokines (e.g., TNF-α, IL-1β, IL-6), chemokines, and reactive oxygen species (ROS), which further exacerbate neuronal apoptosis, blood-brain barrier disruption, and cerebral edema, and aggravate neurological deficits (Fu et al., 2015).

**FIGURE 14:**
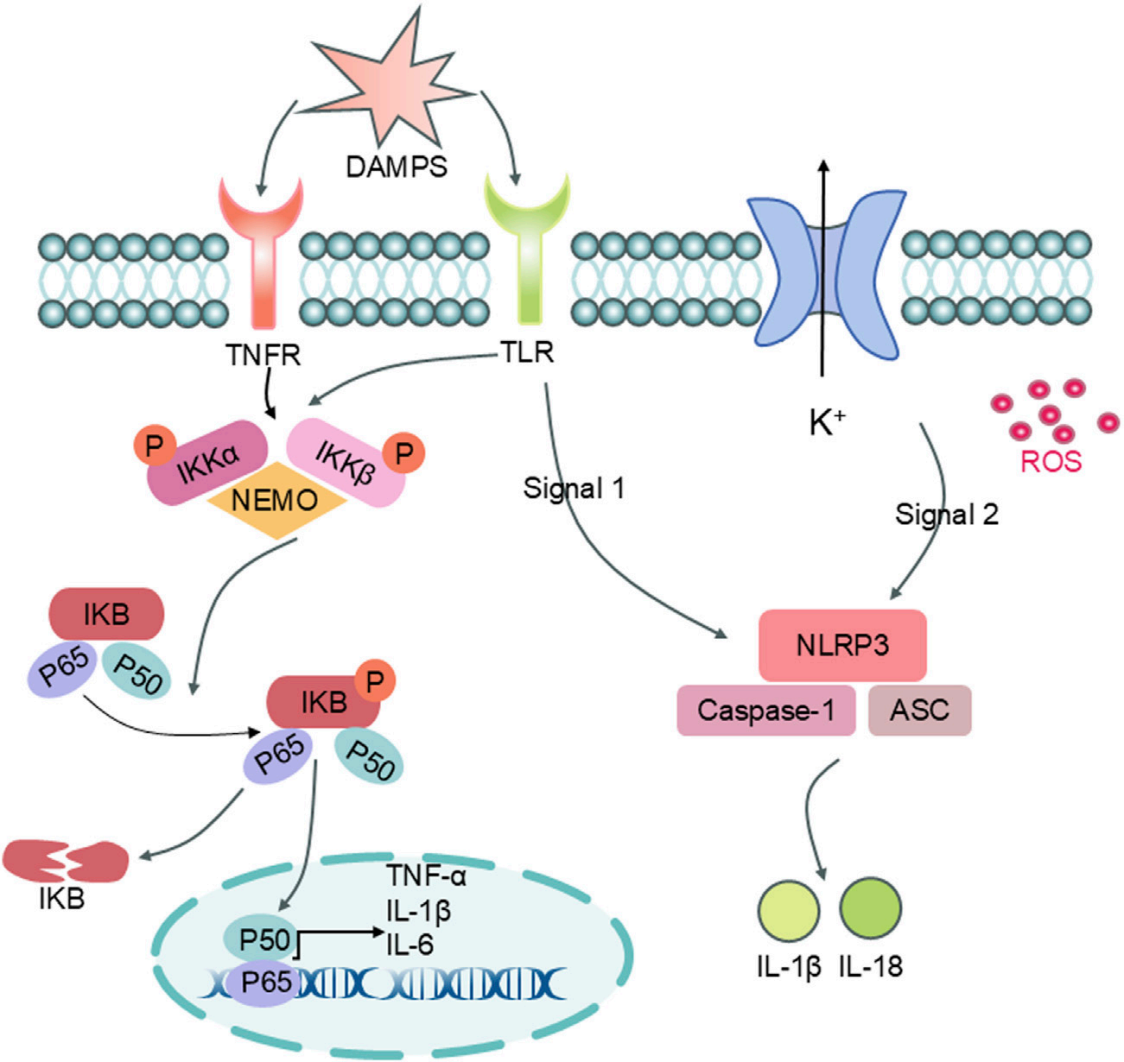
Inflammatory Pathway Diagram (Legend: DAMPs: Damage-Associated Molecular Patterns; TNFR: Tumor Necrosis Factor Receptor; TLR: Toll-like Receptor; IKKα: IκB Kinase alpha; IKKβ: IκB Kinase beta; NEMO: NF-κB Essential Modulator; IKB: Inhibitor of NF-κB; P65: RelA; P50: NF-κB1; TNF-α: Tumor Necrosis Factor-alpha; IL-1β: Interleukin-1 beta; IL-6: Interleukin-6; NLRP3: NOD-, LRR- and pyrin domain-containing protein 3; Caspase-1: Cysteinyl aspartate specific proteinase 1; ASC: Apoptosis-associated speck-like protein containing a CARD; IL-18: Interleukin-18)

The inflammatory cascade after stroke is regulated by several signaling pathways (Figure 14), and Nuclear factor kappa-light-chain-enhancer of activated B cells (NF-κB) is the most classical signaling pathway in the inflammatory response. Under normal conditions, the inhibitory protein IκB binds to NF-κB and retains it in the cytoplasm. When DAMPs activate related receptors, the IκB kinase (IKK) complex is activated, resulting in the degradation of inhibitory protein IκB and the release of NF-κB (mainly in the form of P65/P50 dimer), which moves into the nucleus and promotes the transcription and expression of inflammatory factors (e.g., IL-6, TNF-α, and IL-1β), leading to enhanced inflammatory responses (Guldenpfennig et al., 2023). Excessive activity of NF-κB has been shown to be associated with neurological damage after stroke. has been shown to be closely related to the degree of neurological damage after stroke. Some studies have shown that Ligustilide can inhibit the nuclear displacement of NF-κB, reduce the release of inflammatory factors, and alleviate the inflammatory response (Huang et al., 2021; Liu et al., 2024; Chen et al., 2025).

Activation signaling of the inflammasome NOD-like receptor family pyrin domain-containing 3 (NLRP3) is also an important pathway associated with the inflammatory response. The NLRP3 inflammasome consists of a combination of proteins, mainly NLRP3, ASC (apoptosis-associated speckled-like protein), and Cysteine-aspartic acid protease-1 (caspase-1) (Agostini et al., 2004). Two signals are required for the activation of this pathway, firstly, TLRs are required to mediate the increase in the expression of NLRP3 and pro-IL-1β (Bauernfeind et al., 2009; Franchi et al., 2009); Secondly, when intracellular conditions such as increased ROS, potassium ion efflux, and mitochondrial damage occur, this promotes NLRP3 oligomerization and assembly of inflammatory vesicles, which results in caspase-1 activation and promotes the splitting of pro-IL-1β and pro-IL-18 into mature IL-1β and IL-18, leading to a dramatic inflammatory response (Schmid-Burgk et al., 2016; Shi et al., 2016). The degree of NLRP3 activation after stroke is closely related to the volume of cerebral infarction and inflammatory cell infiltration. In both animal and cellular experiments, it has been confirmed that Ligustilide can inhibit the activation of NLRP3 inflammatory vesicles, reduce the expression of related inflammatory factors, and attenuate ischemic brain injury (Hu et al., 2020; Li et al., 2020).

In addition to these signaling pathways, Ligustilide may also modulate the activation states of microglia. Microglial polarization toward the M1 phenotype contributes to the amplification of neuroinflammation, whereas the M2 phenotype promotes resolution and repair. Ligustilide has been reported to inhibit M1-like activation while promoting M2 polarization, thereby alleviating post-stroke inflammatory injury and supporting neuronal survival (Wang et al., 2010; Peng et al., 2024; Wu et al., 2025).

### 4.5 Anti-apoptotic mechanisms

Programmed cell death in the form of apoptosis plays a crucial role in embryogenesis, immune regulation, and tissue homeostasis, and is tightly controlled by a series of molecular mechanisms. Unlike necrosis, apoptosis is characterized by intact membrane structures and minimal inflammatory response, relying on a tightly controlled cascade of intracellular signaling events and protease activation. Although physiologically beneficial, excessive or aberrant activation of apoptosis can lead to irreversible tissue damage under pathological conditions, particularly in central nervous system (CNS) disorders (Bredesen, 1995).

In IS, apoptosis is one of the predominant forms of secondary brain injury, especially in the ischemic penumbra. Following ischemia, energy failure, excitotoxic glutamate release (Chamorro et al., 2016), intracellular calcium overload (Nakagawa, 2019), and the overproduction of reactive ROS (Szabó et al., 2020) contribute to the initiation of apoptotic pathways in neurons and vascular endothelial cells. These pathological stimuli ultimately result in structural and functional neuronal damage, leading to progressive neuronal loss and exacerbated neurological deficits.

**FIGURE 15:**
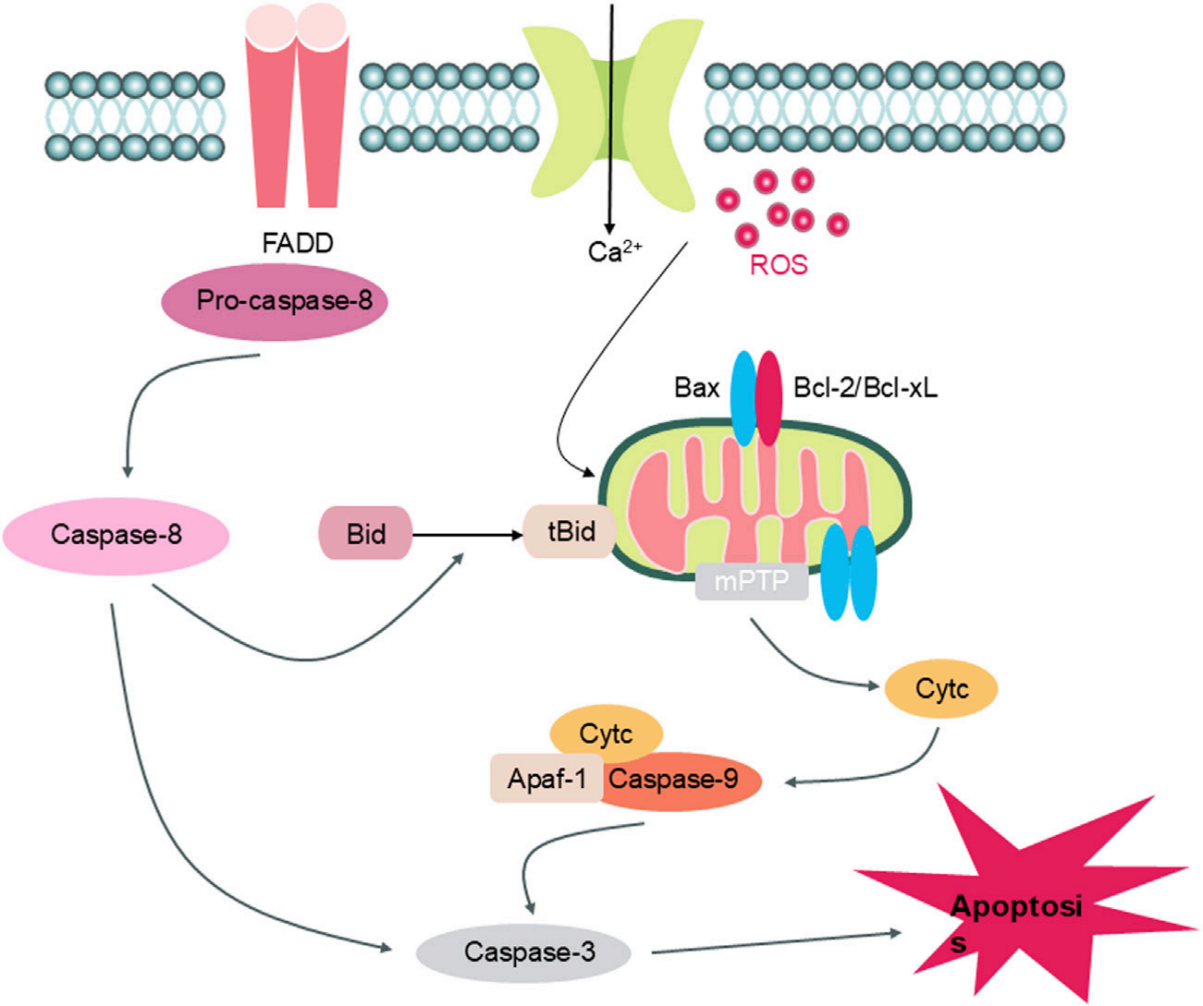
Apoptosis Mechanism Diagram (Legend: FADD: Fas-Associated protein with Death Domain; Pro-caspase-8: Pro-Caspase-8; caspase-8: Cysteinyl aspartate specific proteinase 8; caspase-3: Cysteinyl aspartate specific proteinase 3; ROS: Reactive Oxygen Species; Bax: Bcl-2-associated X protein; Bcl-2: B-cell lymphoma 2; Bcl-xl: B-cell lymphoma (extra large); tBid: Truncated Bid; Bid: BH3 interacting-domain death agonist; mPTP: Mitochondrial Permeability Transition Pore; Cytc: Cytochrome c; Apaf-1: Apoptotic Protease Activating Factor-1; Caspase-9: Cysteinyl aspartate specific proteinase 9).

Apoptotic cell death following cerebral ischemia is mediated through multiple interrelated pathways, primarily comprising the intrinsic (mitochondria-dependent) and extrinsic (death receptor-associated) mechanisms (Figure 15). Among these, the intrinsic mitochondrial cascade is regarded as the predominant and best-characterized route. Energy failure, calcium overload, and elevated oxidative stress contribute to increased mitochondrial membrane permeability, leading to the dissipation of mitochondrial membrane potential and subsequent release of cytochrome c into the cytosol. Once in the cytoplasm, cytochrome c interacts with Apaf-1 and procaspase-9 to assemble the apoptosome, thereby initiating caspase-9 activation and subsequent activation of effector caspase-3, culminating in DNA degradation and cellular disintegration (Bredesen, 1995; Wang et al., 2019). The B-cell lymphoma 2 (Bcl-2) family of proteins serves as a key modulator of this pathway. Pro-apoptotic proteins such as Bcl-2-associated X protein (Bax) and Bcl-2 homologous antagonist killer (Bak) facilitate the opening of the mitochondrial permeability transition pore (mPTP) (Kist and Vucic, 2021), while anti-apoptotic counterparts including Bcl-2 and Bcl-xL act to stabilize mitochondrial function. Ischemia induces an imbalance in these proteins—upregulating Bax and downregulating Bcl-2—resulting in an increased Bax/Bcl-2 ratio, a hallmark of enhanced neuronal apoptotic susceptibility.

The extrinsic death receptor pathway, mediated by transmembrane proteins such as Fas and TNF receptors, represents a critical link between inflammation and cell death (Martin-Villalba et al., 1999). Following stroke, pro-inflammatory cytokines including TNF-α and IL-1β (Cantarella et al., 2014; Ma et al., 2017) activate the Fas/FasL system, recruiting Fas-associated death domain (FADD) protein and procaspase-8 (Velier et al., 1999; Muhammad et al., 2018). Once activated, caspase-8 triggers downstream caspase-3 activation, culminating in apoptotic cell death (Morita-Fujimura et al., 2001). Notably, caspase-8 can also amplify intrinsic apoptosis via the truncated Bid (tBid) pathway, thereby linking both apoptotic routes (Xu et al., 2017).

Previous *in vivo* and *in vitro* studies have shown that Ligustilide significantly attenuates apoptosis by downregulating caspase-9 and caspase-3 expression (Chao and Korsmeyer, 1998; Ferrer and Planas, 2003), modulating the Bax/Bcl-2 ratio, and ultimately suppressing neuronal cell death following ischemic injury (Wu et al., 2020; Peng et al., 2022a; Wu et al., 2022b).

Although the current findings suggest that Ligustilide may exert neuroprotective effects through antioxidant, anti-inflammatory, and anti-apoptotic mechanisms in preclinical models of ischemic stroke, the translational relevance of these mechanisms remains uncertain. A well-recognized challenge in stroke research is the translational gap between rodent models and human clinical outcomes. Many neuroprotective agents that showed promising results in animals have failed in clinical trials, often due to poor reproducibility, limited modeling of comorbidities, and differences in treatment timing, dosing, and outcome assessments. Therefore, while the mechanistic insights gained from these animal studies are valuable, they should be interpreted cautiously and further validated in clinically relevant models and human studies.

## 5 Limitations and future perspectives

This meta-analysis and systematic review provided an in-depth assessment of the therapeutic efficacy of Ligustilide in preclinical models of IS. The results suggest that Ligustilide may exert neuroprotective effects by improving neurological deficits, reducing infarct volume, attenuating inflammatory responses, alleviating oxidative stress, and potentially modulating apoptosis. These findings indicate its potential value in the treatment of stroke. However, the current preclinical evidence has several limitations that warrant cautious interpretation. First, substantial heterogeneity exists among the included studies in terms of experimental design. Variations in animal species, sex, and body weight may influence the pathological features of stroke models and the pharmacological response to treatment.

Nevertheless, the standardized mean differences (SMDs) observed for certain outcomes, particularly infarct volume (SMD = 3.26), appear higher than those typically reported in preclinical neuroprotection studies. Such inflated effect sizes may result from small-sample effects, methodological variability, and differences in outcome measurement scales across studies. To provide a more intuitive reference of the treatment magnitude, raw mean differences (MD ± SD) for infarct volume and neurological function have been included in the supplementary materials (see Supplementary Tables 4, 5). Moreover, small-study bias and within-model heterogeneity could have contributed to the overestimation of pooled effects, a phenomenon commonly observed in preclinical stroke meta-analyses. Therefore, these findings should be interpreted with caution and in light of the limitations inherent to preclinical animal studies.

Furthermore, funnel-plot analyses in preclinical meta-analyses should be interpreted cautiously, as the limited number of animal studies and small sample sizes greatly reduce the power to detect true asymmetry or publication bias.

In addition, modeling approaches differed across studies; distinct ischemic models vary in the severity of ischemia and duration of reperfusion, which may impact the accuracy and comparability of efficacy evaluations. Notably, the included studies employed different animal models, including transient forebrain cerebral ischemia (FCI), MCAO, and reperfusion-based MCAO models. These models differ in their anatomical targets, the extent and duration of ischemic damage, and their representation of clinical stroke subtypes. Such variability may partially account for the observed heterogeneity and limits the direct translatability of the findings to human stroke conditions. Regarding administration, some studies employed intraperitoneal injection, while others used intravenous or oral delivery. Drug dosages and treatment timings also varied and lacked standardization. These inconsistencies may compromise the comparability of outcomes across studies. Second, despite an extensive literature search, only 13 animal studies met the inclusion criteria, resulting in a relatively limited overall sample size. This limitation is especially pronounced for secondary outcomes, where some analyses were based on only two studies, weakening the stability and representativeness of the results. Moreover, part of the numerical data was derived from digitized graphical plots using tools such as WebPlotDigitizer, which may introduce bias due to variations in image quality or coordinate recognition. Furthermore, although animal models can replicate key features of IS pathophysiology, they cannot fully reflect the complex etiology, heterogeneity, and individual variability of human stroke. In addition, most of the included studies did not report the use of blinding procedures during outcome assessment, which could introduce potential detection bias. Whether the beneficial effects observed in animal studies can be translated into clinical efficacy remains to be determined and requires validation in high-quality clinical trials.

Ligustilide is characterized by rapid absorption and broad tissue distribution, including the ability to cross the blood–brain barrier, which underlies its central neuroprotective effects. However, its oral bioavailability is relatively low due to poor stability and extensive first-pass hepatic metabolism. *In vivo* studies have demonstrated that ligustilide undergoes oxidative and reductive biotransformation, with renal and biliary excretion being the primary elimination pathways. Moreover, its chemical structure is inherently unstable and prone to degradation upon exposure to heat, light, or oxygen, posing significant challenges for formulation development and clinical translation. To address these pharmacokinetic limitations, several nanocarrier-based formulation strategies have been explored to enhance the stability, brain targeting, and bioavailability of Ligustilide. For instance (Zhang et al., 2024), developed ligustilide-loaded liposomes, which exhibited improved physicochemical stability and enhanced brain penetration, thereby overcoming some of the inherent instability of the free compound. In addition, intranasal administration of Ligustilide has been investigated; pretreatment in a rat model of cerebral ischemia enabled rapid brain uptake and significant neuroprotective effects, supporting a nose-to-brain delivery route that bypasses first-pass hepatic metabolism (Li et al., 2017). Furthermore, lipid-based nanocarriers, such as solid lipid nanoparticles (SLNs) and nanostructured lipid carriers (NLCs), have been widely studied for the delivery of lipophilic drugs to the brain, offering advantages such as improved permeability, sustained release, and reduced systemic clearance (Costa et al., 2021). The integration of natural compounds with brain-targeted nanocarrier systems thus represents a promising direction for enhancing central nervous system drug delivery (Liu et al., 2025). In terms of safety, comprehensive long-term toxicological evaluations are still lacking. These pharmacokinetic and toxicological limitations warrant careful consideration in future translational research.

Given the limitations of current clinical interventions and the promising yet preliminary findings surrounding Ligustilide, rigorous preclinical synthesis is crucial for guiding future translational and clinical applications. Future studies should aim to standardize experimental parameters, including stroke modeling techniques, drug administration routes, dosages, and outcome measures. Particular attention should be given to the selection and consistent use of stroke models, with detailed reporting of ischemia type, duration, reperfusion status, and outcome assessment timepoints, to enhance reproducibility and translational value. Increasing sample size, conducting multicenter and multispecies studies, and enhancing reproducibility are essential for improving evidence reliability. In addition, further mechanistic investigations and translational preclinical experiments are needed to support the development of Ligustilide as a promising therapeutic candidate for IS.

## Data Availability

The original contributions presented in the study are included in the article/Supplementary Material, further inquiries can be directed to the corresponding authors.

## Glossary

IS: ischemic stroke
HS: hemorrhagic stroke
MDA: Malondialdehyde
GSH: Glutathione
SOD: Superoxide Dismutase
TNF-α: Tumor Necrosis Factor-alpha
ROS: Reactive Oxygen Species
Keap1: Kelch-like ECH-associated protein 1
Nrf2: Nuclear factor erythroid 2-related factor 2
ARE: Antioxidant Response Element
HO-1: Heme Oxygenase-1
NQO1: NAD(P)H Quinone Dehydrogenase 1
GCLC: Glutamate-Cysteine Ligase Catalytic Subunit
ERK: Extracellular signal-regulated kinase
CREB: AMP Response Element-Binding protein
JNK: c-Jun N-terminal Kinase
c-Jun: Jun proto-oncogene
P38: p38 Mitogen-Activated Protein Kinase
ATF-2: Activating Transcription Factor 2
DAMPs: Damage-Associated Molecular Patterns
TNFR: Tumor Necrosis Factor Receptor
TLR: Toll-like Receptor
IKKα: IκB Kinase alpha
IKKβ: IκB Kinase beta
NEMO: NF-κB Essential Modulator
IKB: Inhibitor of NF-κB
P65: RelA
P50: NF-κB1
TNF-α: Tumor Necrosis Factor-alpha
IL-1β: Interleukin-1 beta
IL-6: Interleukin-6
NLRP3: NOD-, LRR- and pyrin domain-containing protein 3
Caspase-1: Cysteinyl aspartate specific proteinase 1
ASC: Apoptosis-associated speck-like protein containing a CARD
IL-18: Interleukin-18 FADD, Fas-Associated protein with Death Domain
Pro-caspase-8: Pro-Caspase-8
caspase-8: Cysteinyl aspartate specific proteinase 8
caspase-3: Cysteinyl aspartate specific proteinase 3
Bax: Bcl-2-associated X protein
Bcl-2: B-cell lymphoma 2
Bcl-xl: B-cell lymphoma (extra large)
tBid: Truncated Bid
Bid: BH3 interacting-domain death agonist
mPTP: Mitochondrial Permeability Transition Pore
Cytc: Cytochrome c
Apaf-1: Apoptotic Protease Activating Factor-1
Caspase-9: Cysteinyl aspartate specific proteinase 9
