# Supplementary material for: Preclinical evidence and mechanistic insights of ligustilide in ischemic stroke: a systematic review and meta-analysis
Source: Front Pharmacol. 2025 Nov 21;16:1666207. doi: 10.3389/fphar.2025.1666207 (PMC12731248; doi:10.3389/fphar.2025.1666207)
Supplement: Supplementary file 1 [file Supplementaryfile1.doc]

**supplementary materials**

PubMed

("ischemic stroke"[MeSH Terms] OR ("ischemic"[All Fields] AND "stroke"[All Fields]) OR "ischemic stroke"[All Fields] OR ("ischemic stroke"[MeSH Terms] OR ("ischemic"[All Fields] AND "stroke"[All Fields]) OR "ischemic stroke"[All Fields] OR ("ischemic"[All Fields] AND "strokes"[All Fields]) OR "ischemic strokes"[All Fields]) OR ("ischemic stroke"[MeSH Terms] OR ("ischemic"[All Fields] AND "stroke"[All Fields]) OR "ischemic stroke"[All Fields] OR ("stroke"[All Fields] AND "ischemic"[All Fields]) OR "stroke ischemic"[All Fields]) OR ("ischemic stroke"[MeSH Terms] OR ("ischemic"[All Fields] AND "stroke"[All Fields]) OR "ischemic stroke"[All Fields] OR ("ischaemic"[All Fields] AND "stroke"[All Fields]) OR "ischaemic stroke"[All Fields]) OR ("ischemic stroke"[MeSH Terms] OR ("ischemic"[All Fields] AND "stroke"[All Fields]) OR "ischemic stroke"[All Fields] OR ("ischaemic"[All Fields] AND "strokes"[All Fields]) OR "ischaemic strokes"[All Fields]) OR ("ischemic stroke"[MeSH Terms] OR ("ischemic"[All Fields] AND "stroke"[All Fields]) OR "ischemic stroke"[All Fields] OR ("stroke"[All Fields] AND "ischaemic"[All Fields]) OR "stroke ischaemic"[All Fields]) OR ("ischemic stroke"[MeSH Terms] OR ("ischemic"[All Fields] AND "stroke"[All Fields]) OR "ischemic stroke"[All Fields] OR ("acute"[All Fields] AND "ischemic"[All Fields] AND "stroke"[All Fields]) OR "acute ischemic stroke"[All Fields]) OR ("ischemic stroke"[MeSH Terms] OR ("ischemic"[All Fields] AND "stroke"[All Fields]) OR "ischemic stroke"[All Fields] OR ("acute"[All Fields] AND "ischemic"[All Fields] AND "strokes"[All Fields]) OR "acute ischemic strokes"[All Fields]) OR ("ischemic stroke"[MeSH Terms] OR ("ischemic"[All Fields] AND "stroke"[All Fields]) OR "ischemic stroke"[All Fields] OR ("ischemic"[All Fields] AND "stroke"[All Fields] AND "acute"[All Fields]) OR "ischemic stroke acute"[All Fields]) OR ("ischemic stroke"[MeSH Terms] OR ("ischemic"[All Fields] AND "stroke"[All Fields]) OR "ischemic stroke"[All Fields] OR ("stroke"[All Fields] AND "acute"[All Fields] AND "ischemic"[All Fields]) OR "stroke acute ischemic"[All Fields]) OR ("ischemic stroke"[MeSH Terms] OR ("ischemic"[All Fields] AND "stroke"[All Fields]) OR "ischemic stroke"[All Fields] OR ("cryptogenic"[All Fields] AND "ischemic"[All Fields] AND "stroke"[All Fields]) OR "cryptogenic ischemic stroke"[All Fields]) OR ("ischemic stroke"[MeSH Terms] OR ("ischemic"[All Fields] AND "stroke"[All Fields]) OR "ischemic stroke"[All Fields] OR ("cryptogenic"[All Fields] AND "ischemic"[All Fields] AND "strokes"[All Fields]) OR "cryptogenic ischemic strokes"[All Fields]) OR ("ischemic stroke"[MeSH Terms] OR ("ischemic"[All Fields] AND "stroke"[All Fields]) OR "ischemic stroke"[All Fields] OR ("ischemic"[All Fields] AND "stroke"[All Fields] AND "cryptogenic"[All Fields]) OR "ischemic stroke cryptogenic"[All Fields]) OR ("ischemic stroke"[MeSH Terms] OR ("ischemic"[All Fields] AND "stroke"[All Fields]) OR "ischemic stroke"[All Fields] OR ("stroke"[All Fields] AND "cryptogenic"[All Fields] AND "ischemic"[All Fields])) OR ("ischemic stroke"[MeSH Terms] OR ("ischemic"[All Fields] AND "stroke"[All Fields]) OR "ischemic stroke"[All Fields] OR ("cryptogenic"[All Fields] AND "embolism"[All Fields] AND "stroke"[All Fields]) OR "cryptogenic embolism stroke"[All Fields]) OR ("ischemic stroke"[MeSH Terms] OR ("ischemic"[All Fields] AND "stroke"[All Fields]) OR "ischemic stroke"[All Fields] OR ("cryptogenic"[All Fields] AND "embolism"[All Fields] AND "strokes"[All Fields])) OR ("ischemic stroke"[MeSH Terms] OR ("ischemic"[All Fields] AND "stroke"[All Fields]) OR "ischemic stroke"[All Fields] OR ("embolism"[All Fields] AND "stroke"[All Fields] AND "cryptogenic"[All Fields])) OR ("ischemic stroke"[MeSH Terms] OR ("ischemic"[All Fields] AND "stroke"[All Fields]) OR "ischemic stroke"[All Fields] OR ("stroke"[All Fields] AND "cryptogenic"[All Fields] AND "embolism"[All Fields])) OR ("ischemic stroke"[MeSH Terms] OR ("ischemic"[All Fields] AND "stroke"[All Fields]) OR "ischemic stroke"[All Fields] OR ("cryptogenic"[All Fields] AND "stroke"[All Fields]) OR "cryptogenic stroke"[All Fields]) OR ("ischemic stroke"[MeSH Terms] OR ("ischemic"[All Fields] AND "stroke"[All Fields]) OR "ischemic stroke"[All Fields] OR ("cryptogenic"[All Fields] AND "strokes"[All Fields]) OR "cryptogenic strokes"[All Fields]) OR ("ischemic stroke"[MeSH Terms] OR ("ischemic"[All Fields] AND "stroke"[All Fields]) OR "ischemic stroke"[All Fields] OR ("stroke"[All Fields] AND "cryptogenic"[All Fields]) OR "stroke cryptogenic"[All Fields]) OR ("ischemic stroke"[MeSH Terms] OR ("ischemic"[All Fields] AND "stroke"[All Fields]) OR "ischemic stroke"[All Fields] OR ("wake"[All Fields] AND "up"[All Fields] AND "stroke"[All Fields]) OR "wake up stroke"[All Fields]) OR (("stroke"[MeSH Terms] OR "stroke"[All Fields] OR "strokes"[All Fields] OR "stroke s"[All Fields]) AND "Wake-up"[All Fields]) OR ("ischemic stroke"[MeSH Terms] OR ("ischemic"[All Fields] AND "stroke"[All Fields]) OR "ischemic stroke"[All Fields] OR ("wake"[All Fields] AND "up"[All Fields] AND "stroke"[All Fields]) OR "wake up stroke"[All Fields]) OR ("ischemic stroke"[MeSH Terms] OR ("ischemic"[All Fields] AND "stroke"[All Fields]) OR "ischemic stroke"[All Fields] OR ("wake"[All Fields] AND "up"[All Fields] AND "strokes"[All Fields]) OR "wake up strokes"[All Fields]) OR ("reperfusion injury"[MeSH Terms] OR ("reperfusion"[All Fields] AND "injury"[All Fields]) OR "reperfusion injury"[All Fields] OR ("reperfusion"[All Fields] AND "injuries"[All Fields]) OR "reperfusion injuries"[All Fields] OR (("injurie"[All Fields] OR "injuried"[All Fields] OR "injuries"[MeSH Subheading] OR "injuries"[All Fields] OR "wounds and injuries"[MeSH Terms] OR ("wounds"[All Fields] AND "injuries"[All Fields]) OR "wounds and injuries"[All Fields] OR "injurious"[All Fields] OR "injury s"[All Fields] OR "injuryed"[All Fields] OR "injurys"[All Fields] OR "injury"[All Fields]) AND "Ischemia-Reperfusion"[All Fields]) OR ("reperfusion injury"[MeSH Terms] OR ("reperfusion"[All Fields] AND "injury"[All Fields]) OR "reperfusion injury"[All Fields] OR ("injury"[All Fields] AND "ischemia"[All Fields] AND "reperfusion"[All Fields]) OR "injury ischemia reperfusion"[All Fields]) OR ("reperfusion injury"[MeSH Terms] OR ("reperfusion"[All Fields] AND "injury"[All Fields]) OR "reperfusion injury"[All Fields] OR ("ischemia"[All Fields] AND "reperfusion"[All Fields] AND "injuries"[All Fields]) OR "ischemia reperfusion injuries"[All Fields]) OR ("reperfusion injury"[MeSH Terms] OR ("reperfusion"[All Fields] AND "injury"[All Fields]) OR "reperfusion injury"[All Fields] OR ("injury"[All Fields] AND "reperfusion"[All Fields]) OR "injury reperfusion"[All Fields]) OR ("ischaemia reperfusion injury"[All Fields] OR "reperfusion injury"[MeSH Terms] OR ("reperfusion"[All Fields] AND "injury"[All Fields]) OR "reperfusion injury"[All Fields] OR ("ischemia"[All Fields] AND "reperfusion"[All Fields] AND "injury"[All Fields]) OR "ischemia reperfusion injury"[All Fields]) OR ("ischaemia reperfusion injury"[All Fields] OR "reperfusion injury"[MeSH Terms] OR ("reperfusion"[All Fields] AND "injury"[All Fields]) OR "reperfusion injury"[All Fields] OR ("ischemia"[All Fields] AND "reperfusion"[All Fields] AND "injury"[All Fields]) OR "ischemia reperfusion injury"[All Fields]) OR ("reperfusion injury"[MeSH Terms] OR ("reperfusion"[All Fields] AND "injury"[All Fields]) OR "reperfusion injury"[All Fields] OR ("reperfusion"[All Fields] AND "damage"[All Fields]) OR "reperfusion damage"[All Fields]) OR ("reperfusion injury"[MeSH Terms] OR ("reperfusion"[All Fields] AND "injury"[All Fields]) OR "reperfusion injury"[All Fields] OR ("damage"[All Fields] AND "reperfusion"[All Fields]) OR "damage reperfusion"[All Fields]) OR ("reperfusion injury"[MeSH Terms] OR ("reperfusion"[All Fields] AND "injury"[All Fields]) OR "reperfusion injury"[All Fields] OR ("reperfusion"[All Fields] AND "damages"[All Fields]) OR "reperfusion damages"[All Fields]))) AND (((("ligustilide"[Supplementary Concept] OR "ligustilide"[All Fields] OR "ligustilides"[All Fields]) AND "E"[All Fields]) AND ("isomerism"[MeSH Terms] OR "isomerism"[All Fields] OR "isomer"[All Fields] OR "isomers"[All Fields])) OR ((("ligustilide"[Supplementary Concept] OR "ligustilide"[All Fields] OR "ligustilides"[All Fields]) AND "Z"[All Fields]) AND ("isomerism"[MeSH Terms] OR "isomerism"[All Fields] OR "isomer"[All Fields] OR "isomers"[All Fields])) OR ("ligustilide"[Supplementary Concept] OR "ligustilide"[All Fields] OR "z ligustilide"[All Fields]))

Web Of Science

Reperfusion Injuries (All Fields) or Injury, Ischemia-Reperfusion (All Fields) or Injury, Ischemia Reperfusion (All Fields) or Ischemia-Reperfusion Injuries (All Fields) or Injury, Reperfusion (All Fields) or Ischemia-Reperfusion Injury (All Fields) or Ischemia Reperfusion Injury (All Fields) or Reperfusion Damage (All Fields) or Damage, Reperfusion (All Fields) or Reperfusion Damages (All Fields) or Ischemic stroke (All Fields) or Ischemic Strokes (All Fields) or Stroke, Ischemic (All Fields) or Ischaemic Stroke (All Fields) or Ischaemic Strokes (All Fields) or Stroke, Ischaemic (All Fields) or Acute Ischemic Stroke (All Fields) or Acute Ischemic Strokes (All Fields) or Ischemic Stroke, Acute (All Fields) or Stroke, Acute Ischemic (All Fields) or Cryptogenic Ischemic Stroke (All Fields) or Cryptogenic Ischemic Strokes (All Fields) or Ischemic Stroke, Cryptogenic (All Fields) or Stroke, Cryptogenic Ischemic (All Fields) or Cryptogenic Embolism Stroke (All Fields) or Cryptogenic Embolism Strokes (All Fields) or Embolism Stroke, Cryptogenic (All Fields) or Stroke, Cryptogenic Embolism (All Fields) or Cryptogenic Stroke (All Fields) or Cryptogenic Strokes (All Fields) or Stroke, Cryptogenic (All Fields) or Wake-up Stroke (All Fields) or Stroke, Wake-up (All Fields) or Wake up Stroke (All Fields) or Wake-up Strokes (All Fields)

AND

ligustilide (All Fields) or Z-ligustilide (All Fields)

EMBASE

reperfusion AND injuries OR (injury, AND 'ischemia reperfusion') OR (injury, AND ischemia AND reperfusion) OR ('ischemia reperfusion' AND injuries) OR 'reperfusion injury' OR ('ischemia reperfusion' AND injury) OR (ischemia AND reperfusion AND injury) OR (reperfusion AND damage) OR (damage, AND reperfusion) OR (reperfusion AND damages) OR (ischemic AND stroke) OR (ischemic AND strokes) OR (stroke, AND ischemic) OR (ischaemic AND stroke) OR (stroke, AND ischaemic) OR (ischaemic AND strokes) OR (acute AND ischemic AND stroke) OR (acute AND ischemic AND strokes) OR (ischemic AND stroke, AND acute) OR (stroke, AND acute AND ischemic) OR (cryptogenic AND ischemic AND stroke) OR (cryptogenic AND ischemic AND strokes) OR (ischemic AND stroke, AND cryptogenic) OR (stroke, AND cryptogenic AND ischemic) OR (cryptogenic AND embolism AND stroke) OR (cryptogenic AND embolism AND strokes) OR (embolism AND stroke, AND cryptogenic) OR (stroke, AND cryptogenic AND embolism) OR (cryptogenic AND stroke) OR (cryptogenic AND strokes) OR (stroke, AND cryptogenic) OR ('wake up' AND stroke) OR (stroke, AND 'wake up') OR (wake AND up AND stroke) OR ('wake up' AND strokes)

AND

'ligustilide'/exp OR ligustilide OR 'z ligustilide'

**Supplementary Figure**


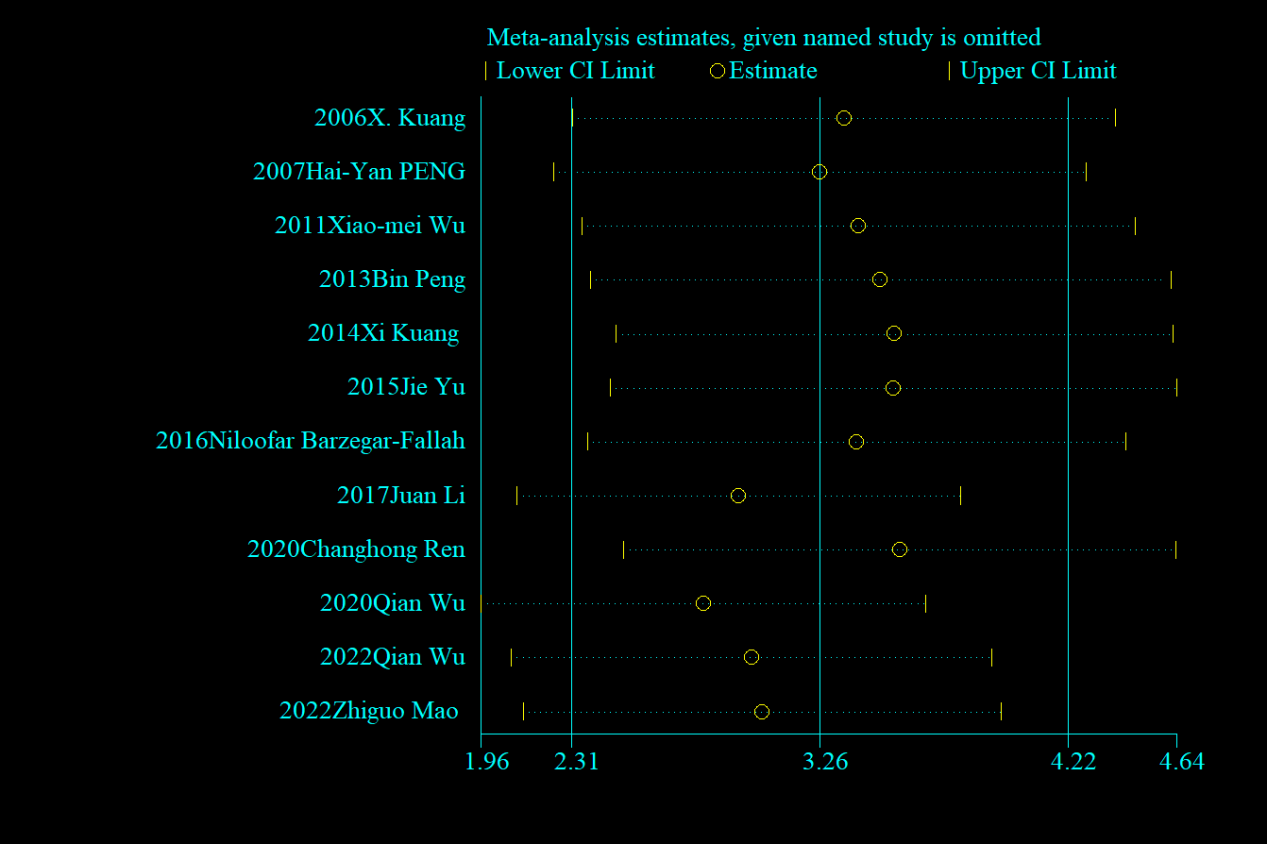


Supplementary Figure 1 Sensitivity analysis - Infarction volume


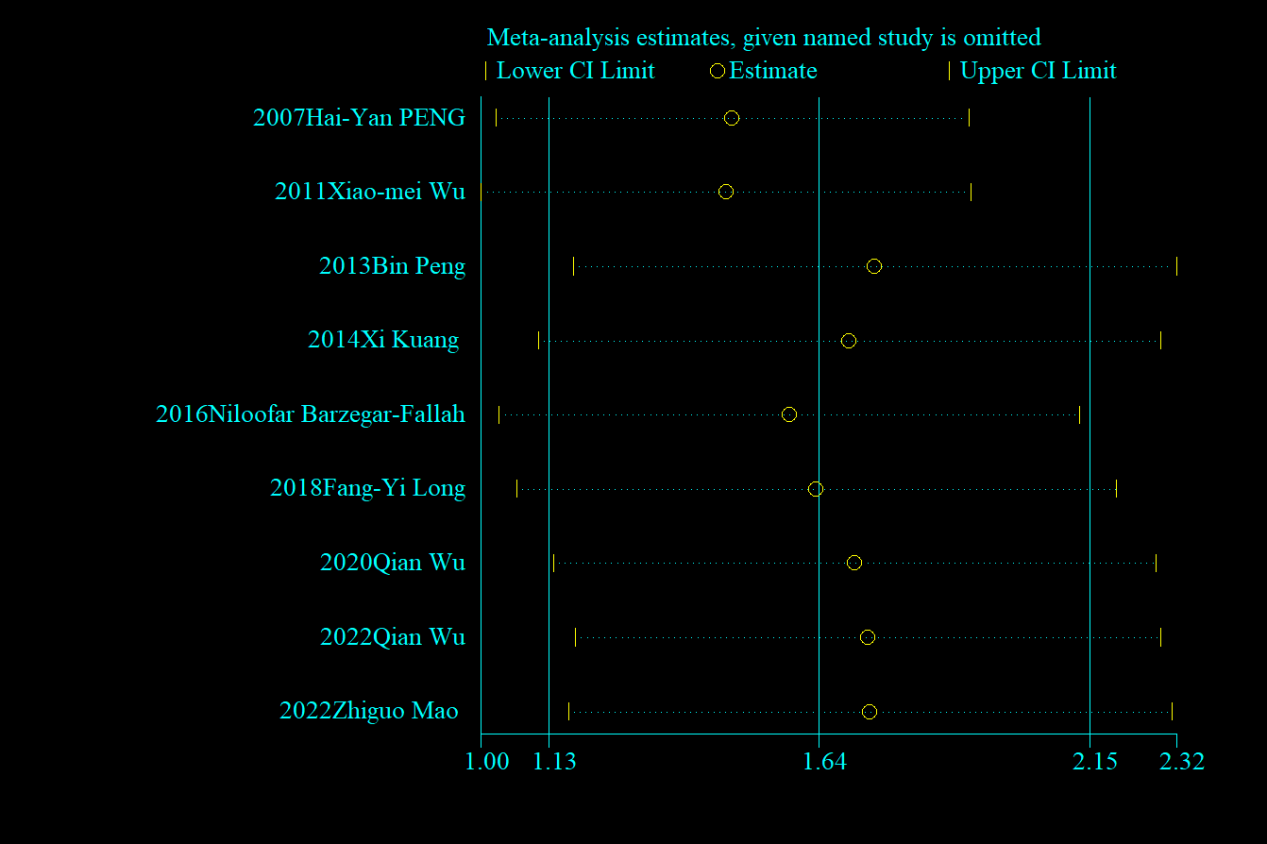


Supplementary Figure 2 Sensitivity analysis - Neurological score


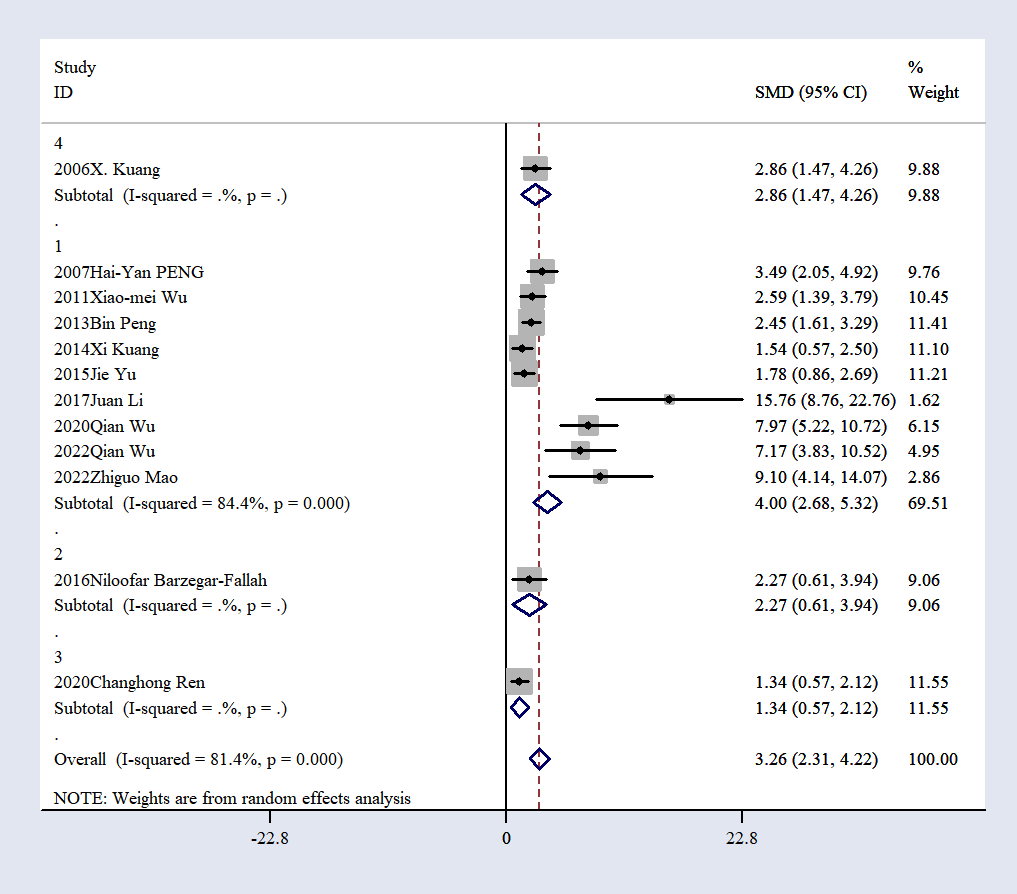


Supplementary Figure 3 Subgroup analysis - classified by species


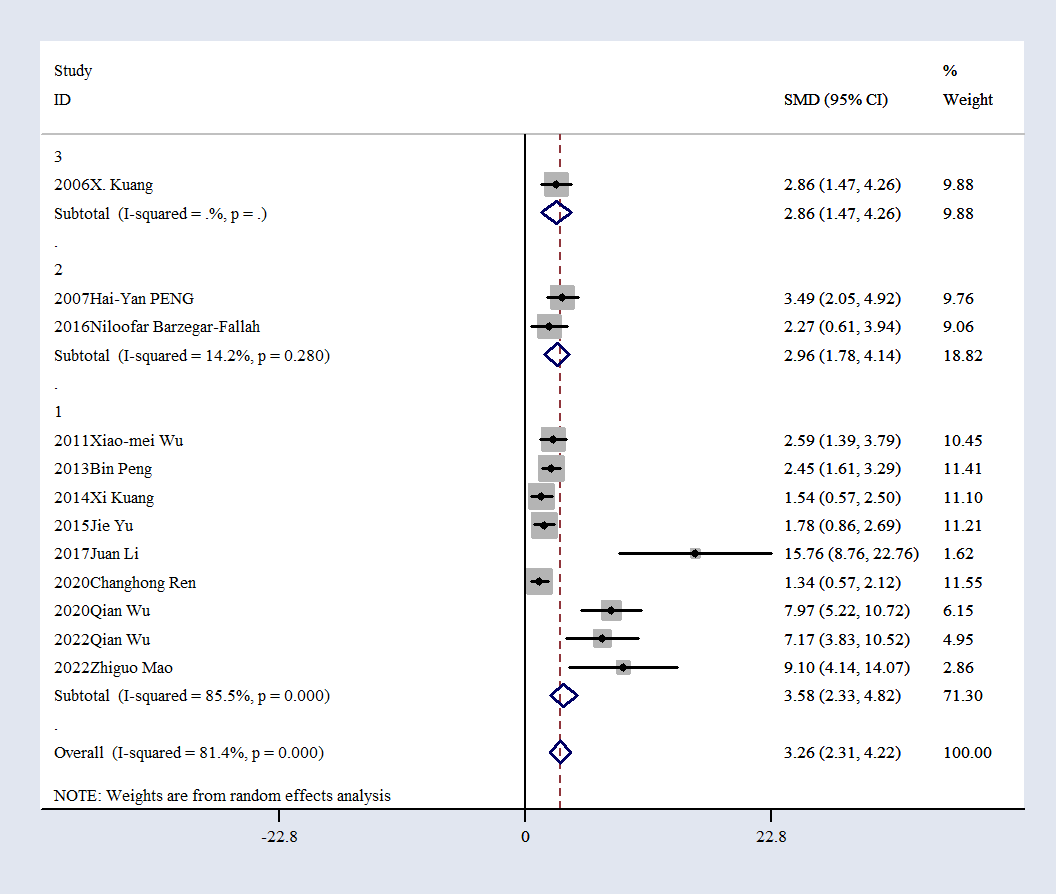


Supplementary Figure 4 Subgroup analysis - by modeling method


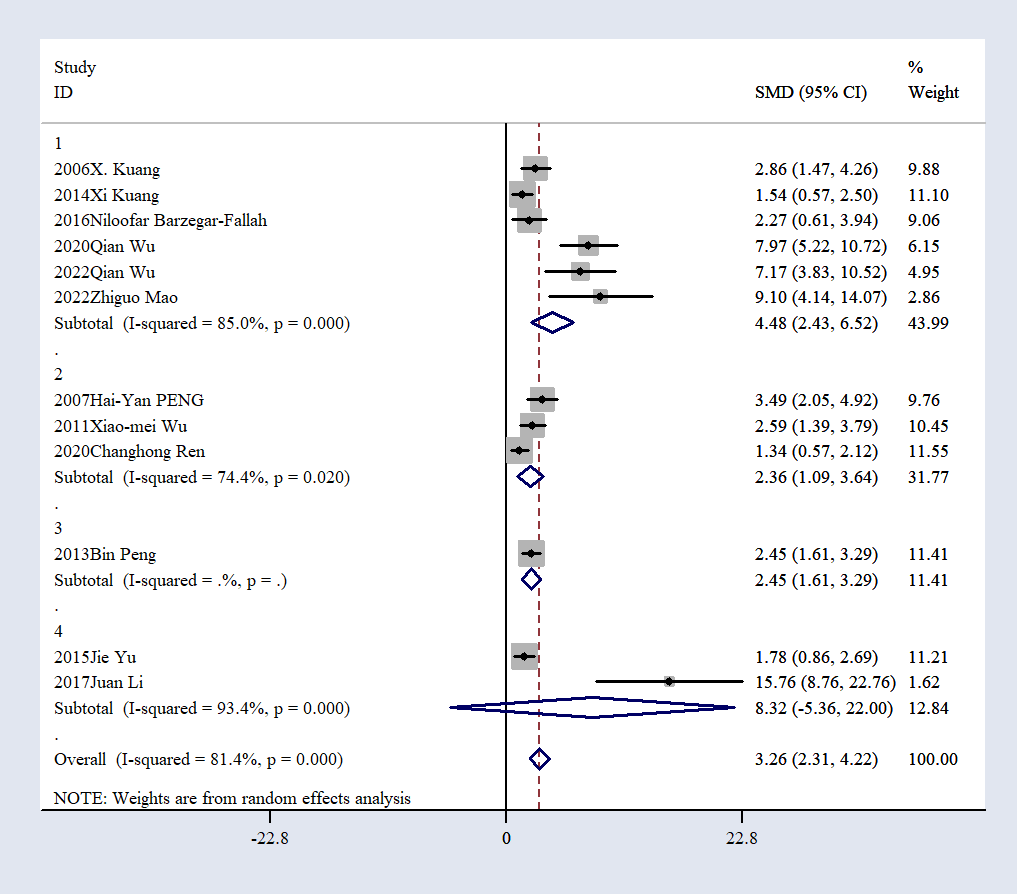


Supplementary Figure 5 Subgroup analysis - by administration method


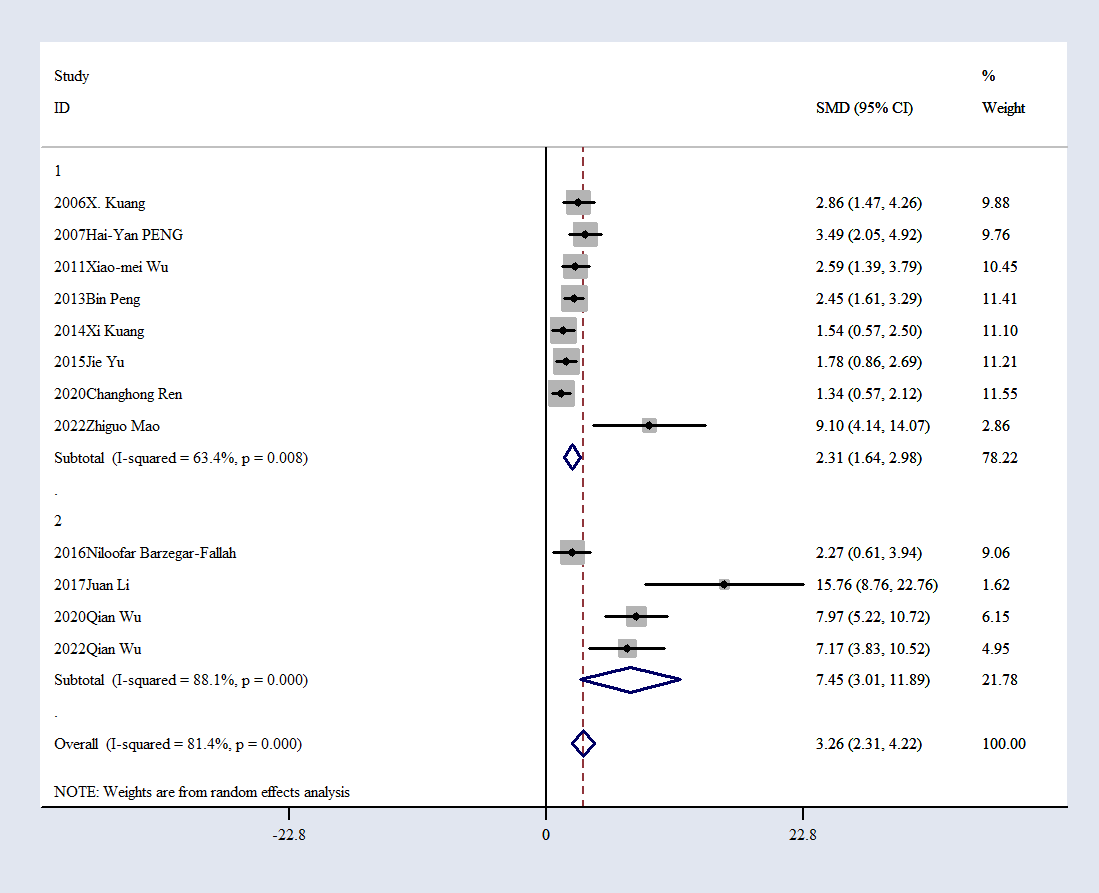
Supplementary Figure 6 Subgroup analysis - Whether to set up multiple-dose groups


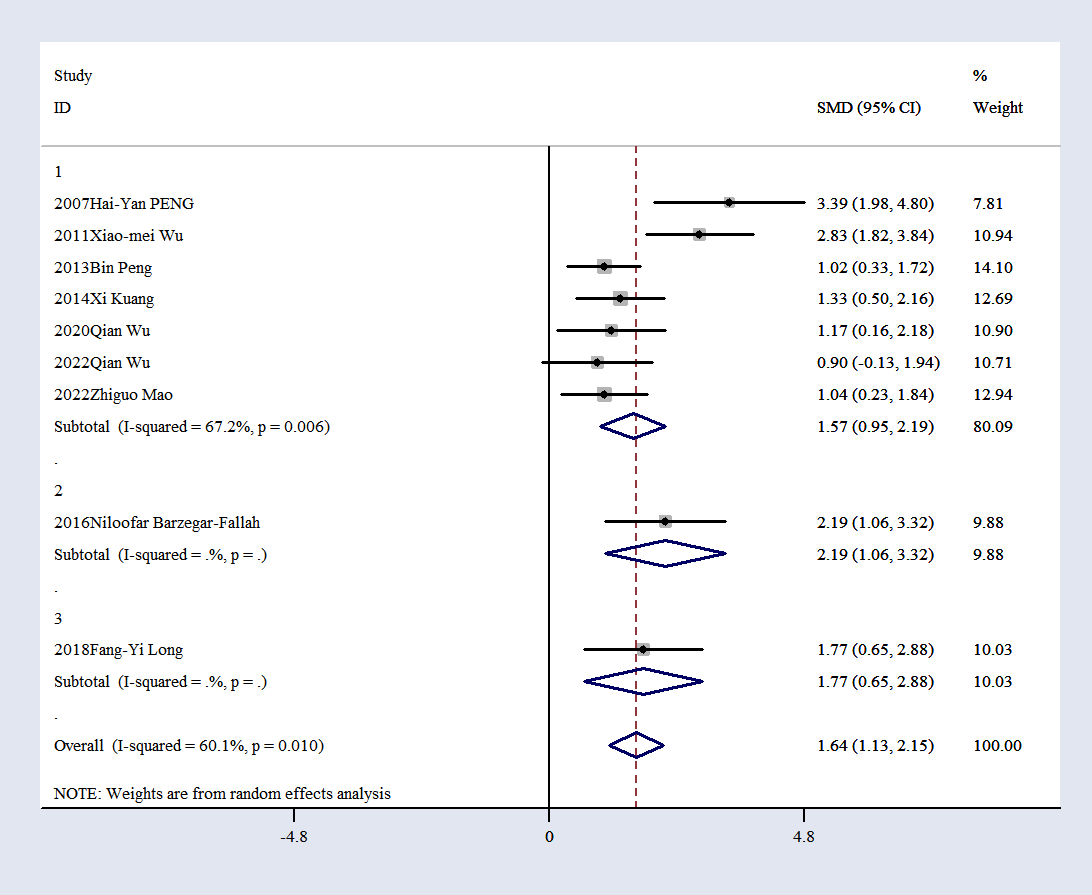
Supplementary Figure 3 Subgroup analysis - classified by species


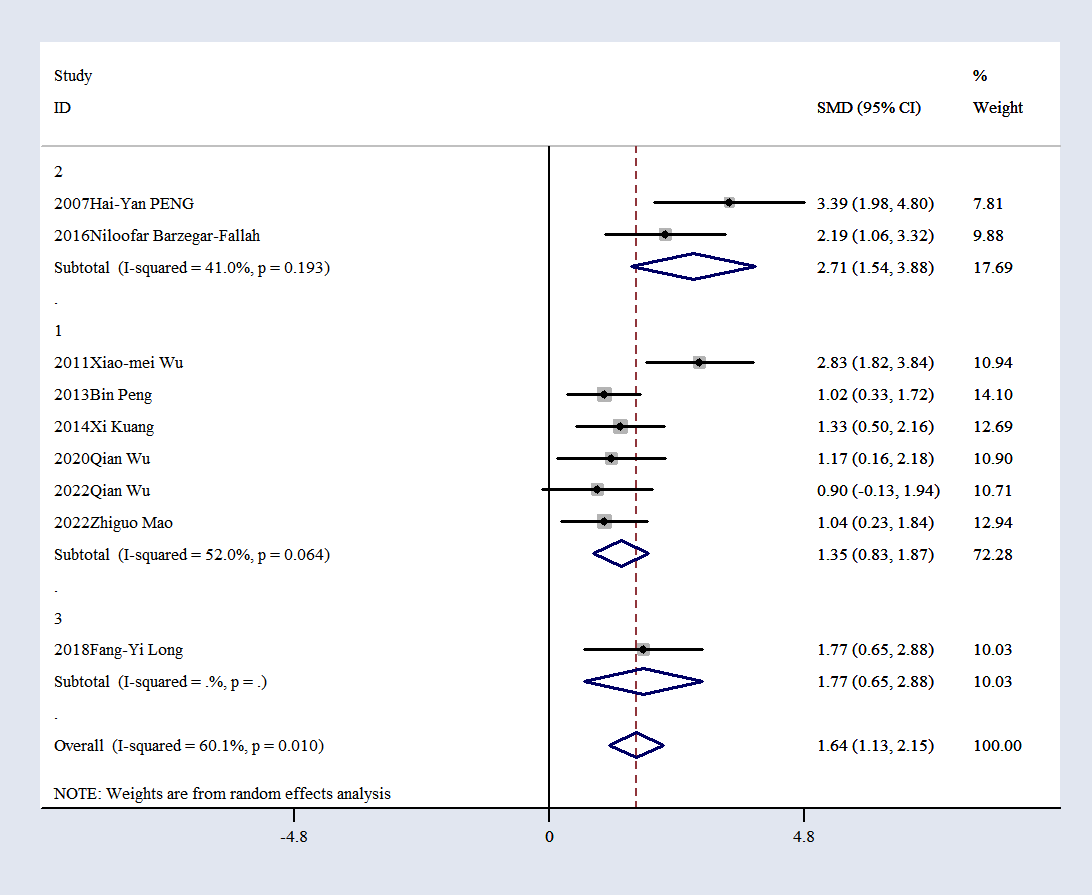
Supplementary Figure 4 Subgroup analysis - by modeling method


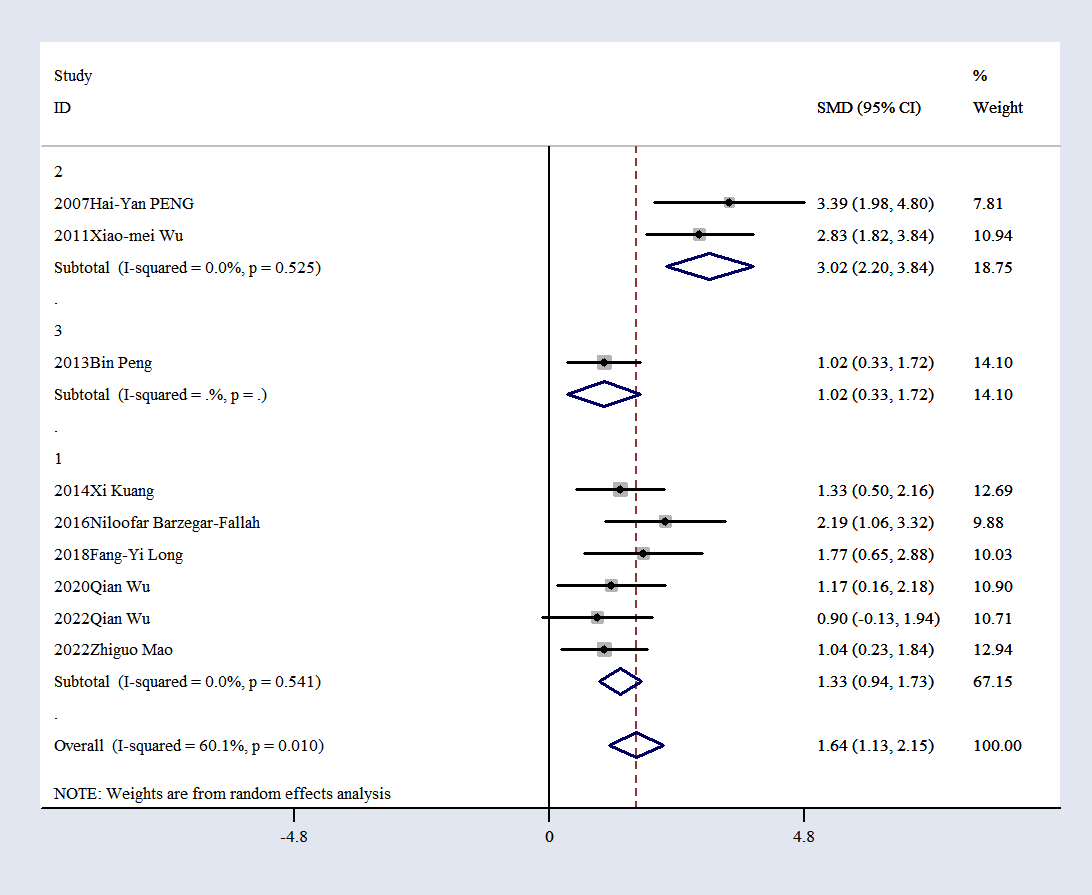
Supplementary Figure 5 Subgroup analysis - by administration method


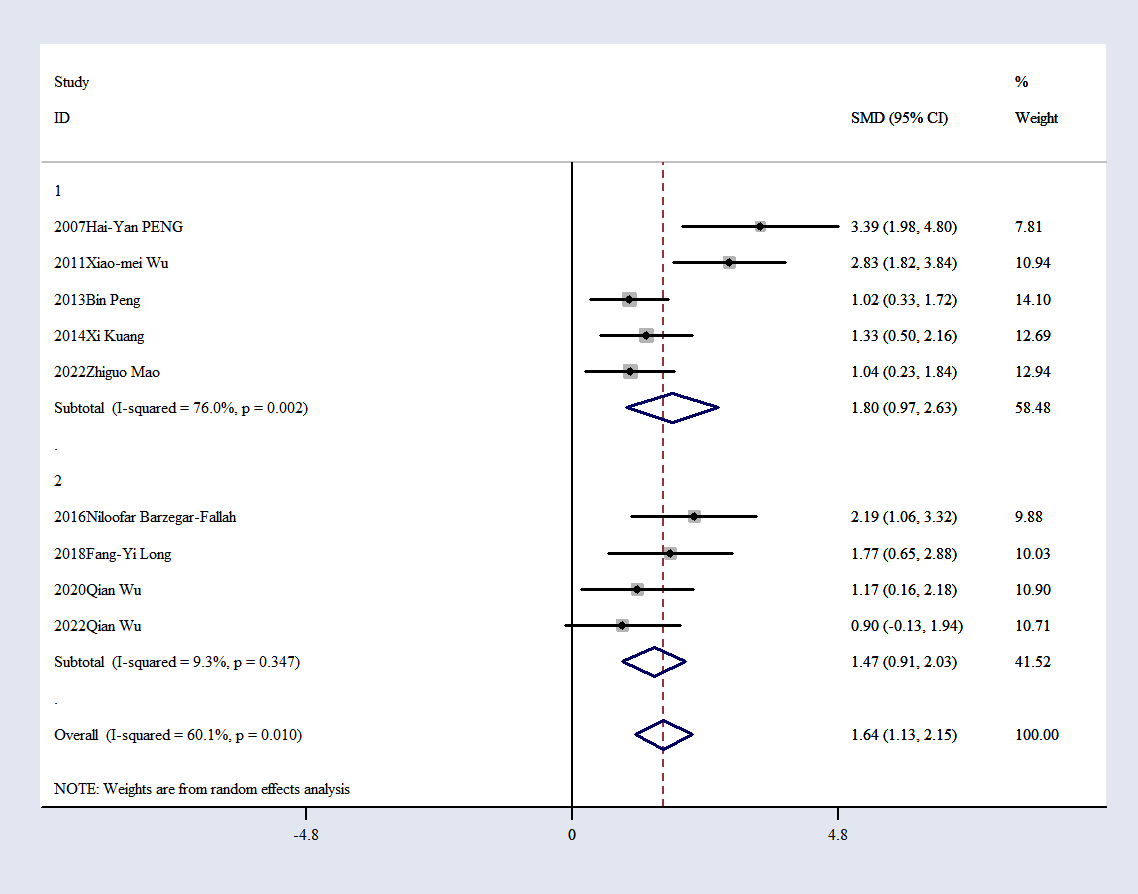
Supplementary Figure 6 Subgroup analysis - Whether to set up multiple-dose groups

**Supplementary Table 1. Subgroup classification table**

| **classify** | species | modeling method | administration method | Whether to set up multiple-dose groups |
| --- | --- | --- | --- | --- |
| **standard** | SD/ICR/Wistar/C57 | FCI/MCAO/MCAO-R/BCCAO | intraperitoneal injection/gavage/tail vein injection/nasal feeding | Yes/No |

**Suppleme**ntary Table 2. Meta-regression of infarction volume

| variable | Coef. (β) | Std. Error | 95% CI | P-value |
| --- | --- | --- | --- | --- |
| animal species | -0.963109 | 1.008506 | [-3.210201- 1.283983] | 0.362 |
| IS modeling methods | -0.9934955 | 1.625577 | [-4.615507- 2.628516] | 0.555 |
| route of administration | 0.0095112 | 1.016017 | [-2.254315- 2.273338] | 0.993 |
| Whether multiple intervention doses exist | 3.779144 | 1.786304 | [-0.20099- 7.759277] | 0.060 |

**Supplementary Table 3.** Meta-regression of neurological scores

| variable | Coef. (β) | Std. Error | 95% CI | P-value |
| --- | --- | --- | --- | --- |
| animal species | 0.1873478 | 0.4591509 | [-0.8983715- 1.273067] | 0.695 |
| IS modeling methods | 0.4969129 | 0.409032 | [-0.4702941- 1.46412] | 0.264 |
| route of administration | 0.1976693 | 0.4107324 | [-0.7735586- 1.168897] | 0.645 |
| Whether multiple intervention doses exist | -0.2824053 | 0.5983788 | [-1.697346- 1.132536] | 0.651 |

**Supplementary Table 4. The raw mean differences (MD ± SD) for infarct volume**

| Study | N_Control | Mean_Control (％) | SD_Control (％) | N_Treatment | Mean_Treatment (％) | SD_Treatment (％) |
| --- | --- | --- | --- | --- | --- | --- |
| 2006X. Kuang | 6 | 22.1 | 2.6 | 12 | 7.2 | 6.03 |
| 2007Hai-Yan PENG | 7 | 21.08 | 1.82 | 14 | 7.04 | 4.71 |
| 2011Xiao-mei Wu | 6 | 31.53 | 2 | 18 | 12.13 | 8.46 |
| 2013Bin Peng | 12 | 44.04 | 2.39 | 33 | 25.61 | 8.62 |
| 2014Xi Kuang | 8 | 16.9 | 5.9 | 16 | 9.85 | 3.82 |
| 2015Jie Yu | 8 | 20.51 | 3.61 | 24 | 13.03 | 4.38 |
| 2016Niloofar Barzegar-Fallah | 5 | 24.43 | 7.23 | 5 | 9.7 | 5.62 |
| 2017Juan Li | 6 | 26.36 | 1.49 | 6 | 8.62 | 0.56 |
| 2020Changhong Ren | 10 | 54.49 | 12.18 | 30 | 37.68 | 12.63 |
| 2020Qian Wu | 10 | 31.1 | 1.13 | 10 | 15.59 | 2.51 |
| 2022Qian Wu | 6 | 37.47 | 2.5 | 6 | 20.51 | 2.22 |
| 2022Zhiguo Mao | 3 | 38.22 | 1.55 | 6 | 15.32 | 2.81 |

**Supplementary Table 5. The raw mean differences (MD ± SD) for neurological score**

| Study | N_Control | Mean_Control | SD_Control | N_Treatment | Mean_Treatment | SD_Treatment |
| --- | --- | --- | --- | --- | --- | --- |
| 2007Hai-Yan PENG | 7 | 3.63 | 0.21 | 14 | 2.2 | 0.49 |
| 2011Xiao-mei Wu | 9 | 3.57 | 0.67 | 27 | 1.59 | 0.71 |
| 2013Bin Peng | 12 | 3 | 0.61 | 33 | 2.12 | 0.93 |
| 2014Xi Kuang | 8 | 2.6 | 0.5 | 32 | 1.8 | 0.62 |
| 2016Niloofar Barzegar-Fallah | 10 | 3.79 | 0.32 | 10 | 2.21 | 0.97 |
| 2018Fang-Yi Long | 10 | 4.69 | 2.44 | 8 | 1.23 | 1.05 |
| 2020Qian Wu | 8 | 1.99 | 0.63 | 10 | 1.31 | 0.54 |
| 2022Qian Wu | 8 | 2.27 | 0.7 | 8 | 1.77 | 0.35 |
| 2022Zhiguo Mao | 10 | 2.29 | 0.65 | 20 | 1.5 | 0.81 |
